# Synthesis, *α*-Glucosidase inhibitory activity and docking studies of Novel Ethyl 1,2,3-triazol-4-ylmethylthio-5,6-diphenylpyridazine-4-carboxylate derivatives

**DOI:** 10.1186/s13065-023-00973-8

**Published:** 2023-06-26

**Authors:** Loghman Firoozpour, Setareh Moghimi, Somayeh Salarinejad, Mahsa Toolabi, Mahdi Rafsanjani, Roya Pakrad, Farzaneh Salmani, Seyed Mohammad Shokrolahi, Seyed Esmail Sadat Ebrahimi, Saeed Karima, Alireza Foroumadi

**Affiliations:** 1 Department of Medicinal Chemistry, Faculty of Pharmacy, Tehran University of Medical Sciences, Tehran, Iran; 2 Drug Design and Development Research Center, The Institute of Pharmaceutical Sciences (TIPS), Tehran University of Medical Sciences, Tehran, Iran; 3 Department of Medicinal Chemistry, School of Pharmacy, Ahvaz Jundishapur University of Medical Sciences, Ahvaz, Iran; 4 Department of Clinical Biochemistry, School of Medicine, Shahid Beheshti University of Medical Sciences (SBMU), Tehran, Iran

**Keywords:** Pyridizine, *α*-Glucosidase, Click reaction, Triazole, Copper iodide, Diabetes

## Abstract

In this work, a novel series of pyridazine-triazole hybrid molecules were prepared and evaluated as inhibitors of rat intestinal *α*-glucosidase enzyme. Amongst all newly synthesized compounds, **10k** showed good inhibition in the series with IC_50_ value of 1.7 µM which is 100 folds stronger than positive control, acarbose. The cytotoxicity revealed that this compound is not toxic against normal cell line, HDF. The docking studies showed that triazole ring plays an important role in the binding interactions with the active site. The insertion of compound **10k ** into the active pocket of *α*-glucosidase and formation of hydrogen bonds with Leu677 was observed from docking studies. The kinetic studies revealed that this compound has uncompetitive mode of inhibition against *α*-glucosidase enzyme.

**Supplementary Information:**

The online version contains supplementary material available at 10.1186/s13065-023-00973-8.

## Introduction

Diabetes Mellitus (DM) is considered as a progressive hormonic and metabolic disorder of endocrine system which is characterized by the body’s loss of control over blood sugar (glucose) [1]. According to the WHO report, 422 million people worldwide [2] are suffering from diabetes which is associated with increased risks of quite a few micro- and macro-vascular complications such as stroke, retinopathy, neuropathy, nephropathy, coronary artery disease, hypertension and peripheral vascular disease [3–6]. This disease is divided into type 1 diabetes mellitus [T1DM] (resulted from insulin deficiency) and type 2 diabetes mellitus [T2DM] (resulted from resistance towards insulin). Type 2 is the most common type and accounts for 90% of all diabetic patients. Most of the medical approaches are focused on the reduction of the postprandial glucose (PG) level in blood. Current treatment approaches include oral anti-diabetic drugs such as sulfonylureas, thiazolidinediones, metformin, *α*-glucosidase inhibitors and glycosurics. *α*-Glucosidase (EC3.2.1.20) belongs to a glycoside hydrolase enzyme releasing monosaccharides through the lysis of *α*-glucopyranoside bond in saccharide polymer, oligosaccharides, and disaccharides from the non-reducing portion of the oligomeric substrate [7]. Inhibiting the digestion and absorption of carbohydrates by using *α*-glucosidase inhibitors slows down carbohydrate digestion, stabilizes blood glucose level and consequently prevents hyperglycemia in diabetic patients. Three glucosidase inhibitors have been introduced to the market and all are carbohydrate mimics. Acarbose, voglibose, and miglitol have been used in the treatment of Type 2 diabetes (T2M) by inhibiting the activity of *α*-glucosidase and consequently the formation of glucose in the small intestine [8–12].

Heterocyclic rings along with sugar-mimic compounds have been emerged as privileged cores in inhibiting glucosidase [13]. Amongst diverse array of heterocyclic cores, nitrogen-containing rings have found a unique place as *α*-glucosidase inhibitors [14–17] and medicinal chemists are still exploring for new molecules containing these valuable pharmacophores. Since decades, pyridazines and its related compounds have attracted great attention because of their therapeutic importance. The literature survey revealed that this heterocyclic core is present in quite a few number of compounds with different pharmacological properties [18–26].

Click reaction, articulated by Sharpless [27, 28], has been considered as a reliable and practical strategy to improve inefficiencies and slownesses of conventional drug discovery. This method provides a facile and revolutionary approach to the invention of drug-like molecules and completion of combinatorial libraries without the need to professional skill and equipment. In addition, this reaction has provided a powerful way to assemble molecules with well-defined biological functions and proteomic applications through making carbon-heteroatom-carbon bond.

The copper-mediated azide–alkyne cycloaddition is a standard and ideal method to achieve triazole ring as a linker and functional moiety. This useful pharmacophore is one of the most important five-membered heterocyclic rings which could be easily synthesized with no sensitivity to water and oxygen and the need for purification techniques. This valuable scaffold also found in various drugs namely antifungal drugs and bioactive compounds with antibacterial, antiviral, and anti-HIV properties [29–31].

**Fig. 1:**
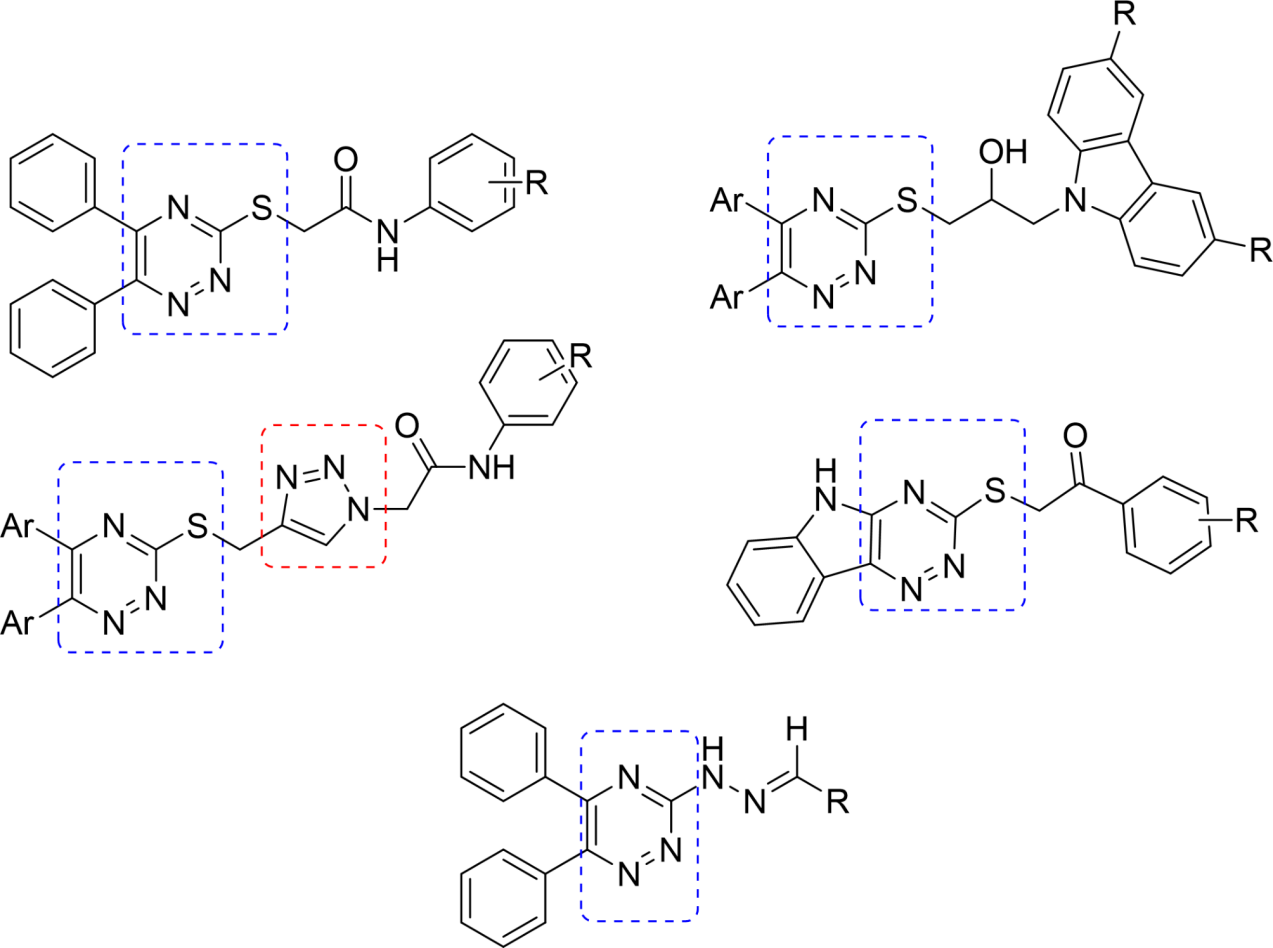
Chemical structures of triazine-containing molecules with *α*-glucosidase inhibitory activity

In recent years, we identified various heterocyclic cores as potential *α*-glucosidase inhibitors [32–35]. Keeping in mind that still there is an urgent need to develop lead candidates, we decided to synthesize novel pyridazine-containing compounds considering the bioisosteric relationship between pyridazine and triazine and the number of triazine-containing compounds with *α*-glucosidase inhibitory activity (Fig. 1) [36–41]. Triazoles were also reported in the literature as inhibitors of *α*-glucosidase enzyme [42]. In this regard, we combined both these cores in one molecule and evaluated their inhibitory activities against *α*-glucosidase enzyme along with kinetic, docking and cytotoxic studies.

## Experimental section

### Chemistry

#### Synthesis of hydrazono-1,2-diphenylethanone (2)

A solution of benzil and hydrazine hydrate in methanol was heated at reflux temperature for 15 min. Then, the reaction mixture was cooled to room temperature and the white solid was collected by filtration, washed with cold methanol and dried [43].

#### Synthesis of ethyl 3-oxo-5,6-diphenyl-2,3-dihydropyridazine-4-carboxylate (3)

A suspension of sodium (0.05 mol) in 200 mL ethanol was chilled to 0 ^o^C and after 15 min, diethyl malonate (0.075 mol) and compound **2** (0.05 mol) were added to the mixture. The mixture was refluxed for 3 h. Upon solvent removal, the residue was acidified with HCl (1 N). The resultant solid was collected and washed with water.

#### Synthesis of ethyl 3-mercapto-5,6-diphenylpyridazine-4-carboxylate (4)

Lawesson’s reagent (10 mmol) was added to the mixture of compound **3** (20 mmol) in toluene (150 mL). The mixture was stirred at reflux temperature for 18 h. After cooling, the solid was separated and washed with toluene, dried and recrystallized from petroleum ether/ethyl acetate.

#### Synthesis of ethyl 5,6-diphenyl-3-(prop-2-yn-1-ylthio)pyridazine-4-carboxylate (5)

Propargyl bromide (12 mmol) was added to the mixture of compound **4** (10 mmol) and K_2_CO_3_ (10 mmol) in DMF (10 mL), and the mixture was stirred at 50 ^o^C. Upon completion, the reaction was stopped with ice/water mixture and the solid was filtered and washed with water.

#### Synthesis of 2-chloro-*N*-arylacetamide (8)

Chloroacetyl chloride (10 mmol) was added to the ice-cooled solution of aromatic amines (10 mmol) and triethylamine (12 mmol) in 1,2-dichloroethane (20 mL). After 6 h stirring at room temperature, petroleum ether was added to the mixture and the solid was filtered and recrystallized from ethanol.

#### Synthesis of 2-azido-*N*-phenylacetamide (9)

A solution of compound 8 (10 mmol) and sodium azide (15 mmol) in dimethyl sulfoxide (20 mL) was stirred at room temperature. Upon completion, indicated by TLC, The reaction mixture was poured into ice/water and the solid was filtered and recrystallized from petroleum ether/ethyl acetate.

#### Synthesis of target compounds (10a-r)

To the suspension of compound **9 **(1 mmol) and compound 5 (1 mmol) in DMF (10 mL), CuI (10 mol %) and triethylamine (1 mmol) was added. The mixture was stirred at room temperature until TLC indicated the disappearance of starting materials. The reaction mixture was poured into ice/water and the solid was collected and washed with water. The solid was recrystallized from ethanol.

#### Ethyl 3-(((1-(2-oxo-2-(phenylamino)ethyl)-1*H*-1,2,3-triazol-4-yl)methyl)thio)-5,6-diphenylpyridazine-4-carboxylate (10a)

Yield (65%, 0.36 g); Dark Yellow solid; M.p. = 147–149 °C; IR (KBr, cm^-1^): 3611, 1730 (C = O), 1652, 1435, 1341, 1160; ^1^ H NMR (DMSO-*d*_6_, 500 MHz): δ 0.86 (t, *J* = 7.1 Hz, 3 H), 4.03 (q, *J* = 7.2 Hz, 2 H), 4.79 (s, 2 H), 5.32 (s, 2 H), 7.07 (t, *J* = 8.0 Hz, 1 H), 7.13 (d, *J* = 8.1 Hz, 2 H), 7.25–7.32 (m, 10 H), 7.56 (d, *J* = 8.3 Hz, 2 H), 8.13 (s, 1 H), 10.45 (s, 1 H); ^13^ C NMR (DMSO-*d*_6_, 100 MHz): δ 13.4, 24.5, 52.9, 62.0, 116.5, 119.2, 124.7, 127.3, 128.3, 128.6 128.8, 129.2, 129.5, 131.2, 134.0, 135.0, 136.2, 138.3, 138.5, 142.0, 155.1, 157.5, 164.1, 166.3; Anal. Calcd. for C_30_H_26_N_6_O_3_S: C, 65.44; H, 4.76; N, 15.26. Found: C, 65.20; H, 4.57; N, 14.98; ESI-MS *m/z*: 550.2 [M]^+^.

#### Ethyl 3-(((1-(2-oxo-2-(m-tolylamino)ethyl)-* H*-1,2,3-triazol-4-yl)methyl)thio)-5,6-diphenylpyridazine-4-carboxylate (10b)

Yield (71%, 0.40 g); Dark Yellow solid; m.p. = 171–173 °C; IR (KBr, cm^-1^): 3623, 1717 (C = O), 1638, 1422, 1319, 1195; ^1^ H NMR (DMSO-*d*_6_, 300 MHz): δ 0.87 (t, *J* = 6.9 Hz, 3 H), 2.25 (s, 3 H), 4.03 (q, *J* = 7.2 Hz, 2 H), 4.79 (s, 2 H), 5.30 (s, 2 H), 7.19–7.30 (m, 14 H), 8.12 (s, 1 H), 10.37 (s, 1 H); ^13^ C NMR (DMSO-*d*_6_, 75 MHz): δ 13.3, 21.1, 24.7, 52.2, 62.1, 116.4, 119.7, 124.4, 127.8, 128.26, 128.33, 128.6, 128.8, 128.9, 129.0, 129.6, 130.4, 133.6, 135.2, 136.0, 138.1, 138.3, 142.4, 155.4, 157.3, 164.0, 166.4; Anal. Calcd. for C_31_H_28_N_6_O_3_S: C, 65.94; H, 5.00; N, 14.88. Found: C, 65.60; H, 4.72; N, 14.59; ESI-MS *m/z*: 564.2 [M]^+^.

#### Ethyl 3-(((1-(2-oxo-2-(p-tolylamino)ethyl)-1*H*-1,2,3-triazol-4-yl)methyl)thio)-5,6-diphenylpyridazine-4-carboxylate (10c)

Yield (70%, 0.39 g); Dark Yellow solid; m.p. = 207–209 °C; IR (KBr, cm^-1^): 3617, 1720 (C = O), 1642, 1442, 1330, 1177; ^1^ H NMR (DMSO-*d*_6_, 500 MHz): δ 0.89 (t, *J* = 7.1 Hz, 3 H), 2.26 (s, 3 H), 4.05 (q, *J* = 7.0 Hz, 2 H), 4.81 (s, 2 H), 5.31 (s, 2 H), 7.12–7.16 (m, 4 H), 7.27–7.36 (m, 8 H), 7.45–7.48 (m, 2 H), 8.14 (s, 1 H), 10.37 (s, 1 H); ^13^ C NMR (DMSO-*d*_6_, 125 MHz): 13.2, 21.0, 24.6, 52.3, 62.0, 116.5, 119.8, 125.0, 126.9, 127.3, 127.7, 128.5, 128.9, 129.1, 129.7, 130.2, 132.9, 135.0, 136.1, 138.4, 142.2, 155.0, 157.1, 164.1, 166.2; Anal. Calcd. for C_31_H_28_N_6_O_3_S: C, 65.94; H, 5.00; N, 14.88. Found: C, 66.20; H, 4.73; N, 15.11; ESI-MS *m/z*: 565.1 [M + H]^+^.

#### Ethyl 3-(((1-(2-((4-isopropylphenyl)amino)-2-oxoethyl)-1*H*-1,2,3-triazol-4-yl)methyl)thio)-5,6-diphenylpyridazine-4-carboxylate (10d)

Yield (68%, 0.40 g); Dark Yellow solid; m.p. = 183–185 °C; IR (KBr, cm^-1^): 3611, 1719 (C = O), 1652, 1441, 1325, 1178; ^1^ H NMR (DMSO-*d*_6_, 500 MHz): δ 0.83–0.90 (m, 9 H), 2.56–2.58 (m, 1 H), 4.03 (q, *J* = 7.0 Hz, 2 H), 4.81 (s, 2 H), 5.33 (s, 2 H), 7.13–7.18 (m, 2 H), 7.29–7.37 (m, 12 H), 8.16 (s, 1 H), 10.53 (s, 1 H); ^13^ C NMR (DMSO-*d*_6_, 125 MHz): δ 13.8, 24.3, 25.2, 33.6, 52.6, 62.6, 115.9, 116.0, 121.5, 126.0, 127.1, 128.3, 128.8, 129.1, 129.3, 129.5, 130.0, 130.1, 130.9, 134.1, 135.2, 136.5, 156.0, 157.9, 164.5, 164.6; Anal. Calcd. for C_33_H_32_N_6_O_3_S: C, 66.87; H, 5.44; N, 14.18. Found: C, 66.57; H, 5.12; N, 13.85; ESI-MS *m/z*: 592.2 [M]^+^.

#### Ethyl 3-(((1-(2-((3-methoxyphenyl)amino)-2-oxoethyl)-1*H*-1,2,3-triazol-4-yl)methyl)thio)-5,6-diphenylpyridazine-4-carboxylate (10e)

Yield (72%, 0.41 g); Dark Yellow solid; m.p. = 155–157 °C; IR (KBr, cm^-1^): 3622, 1718 (C = O), 1639, 1451, 1333, 1175; ^1^ H NMR (DMSO-*d*_6_, 300 MHz): δ 0.87 (t, *J* = 7.1 Hz, 3 H), 3.70 (s, 3 H), 4.06 (q, *J* = 7.0 Hz, 2 H), 4.79 (s, 2 H), 5.31 (s, 2 H), 6.64 (d, *J* = 6.8 Hz, 1 H), 7.07–7.32 (m, 13 H), 8.13 (s, 1 H), 10.44 (s, 1 H); ^13^ C NMR (DMSO-*d*_6_, 75 MHz): δ 13.3, 24.7, 52.2, 55.0, 62.1, 104.9, 109.2, 111.4, 127.8, 128.3, 128.34, 128.58, 128.80, 128.86, 128.97, 129.6, 130.4, 133.6, 135.2, 136.0, 139.5, 142.4, 155.4, 157.3, 159.5, 164.0, 164.6; Anal. Calcd. for C_31_H_28_N_6_O_4_S: C, 64.12; H, 4.86; N, 14.47. Found: C, 63.88; H, 4.57; N, 14.72; ESI-MS *m/z*: 581.2 [M + H]^+^.

#### Ethyl 3-(((1-(2-((4-methoxyphenyl)amino)-2-oxoethyl)-1*H*-1,2,3-triazol-4-yl)methyl)thio)-5,6-diphenylpyridazine-4-carboxylate (10f)

Yield (66%, 0.38 g); Dark Yellow solid; m.p. = 230–232 °C; IR (KBr, cm^-1^): 3601, 1724 (C = O), 1644, 1439, 1327, 1180; ^1^ H NMR (DMSO-*d*_6_, 300 MHz): δ 0.87 (t, *J* = 7.1 Hz, 3 H), 3.70 (s, 3 H), 4.03 (q, *J* = 7.0 Hz, 2 H), 4.79 (s, 2 H), 5.29 (s, 2 H), 6.88 (d, *J* = 8.1 Hz, 2 H), 7.07–7.32 (m, 10 H), 7.49 (d, *J* = 6.8 Hz, 2 H), 8.13 (s, 1 H), 10.33 (s, 1 H); ^13^ C NMR (DMSO-*d*_6_, 75 MHz): δ 13.3, 24.7, 52.1, 55.1, 62.0, 113.9, 120.7, 125.4, 127.8, 128.3, 128.34, 128.8, 128.85, 129.6, 130.4, 131.4, 133.56, 133.62, 135.2, 136.0, 142.3, 155.5, 157.3, 163.6, 164.0; Anal. Calcd. for C_31_H_28_N_6_O_4_S: C, 64.12; H, 4.86; N, 14.47. Found: C, 63.90; H, 4.52; N, 14.74; ESI-MS *m/z*: 580.1 [M]^+^.

#### Ethyl 3-(((1-(2-((4-fluorophenyl)amino)-2-oxoethyl)-1*H*-1,2,3-triazol-4-yl)methyl)thio)-5,6-diphenylpyridazine-4-carboxylate (10 g)

Yield (79%, 0.45 g); Dark Yellow solid; m.p. = 211–213 °C; IR (KBr, cm^-1^): 3616, 1731 (C = O), 1639, 1442, 1332, 1191; ^1^ H NMR (DMSO-*d*_6_, 500 MHz): δ 0.89 (t, *J* = 7.1 Hz, 3 H), 4.06 (q, *J* = 7.0 Hz, 2 H), 4.81 (s, 2 H), 5.33 (s, 2 H), 7.15–7.17 (m, 2 H), 7.27–7.32 (m, 10 H), 7.64–7.66 (m, 2 H), 8.16 (s, 1 H), 10.82 (s, 1 H); ^13^ C NMR (DMSO-*d*_6_, 125 MHz): δ, 13.8, 25.2, 52.6, 62.6, 115.9 (d, *J* = 21 Hz), 121.5 (d, *J* = 8 Hz), 128.2, 128.3, 128.8, 129.1, 129.3, 129.5, 129.6, 129.7, 130.1, 130.9, 134.1, 135.2, 136.5, 156.0, 157.9, 162.3 (d, *J* = 248 Hz), 164.5, 165.6; Anal. Calcd. for C_30_H_25_FN_6_O_3_S: C, 63.37; H, 4.43; N, 14.78. Found: C, 63.02; H, 4.69; N, 15.01; ESI-MS *m/z*: 568.2 [M]^+^.

#### Ethyl 3-(((1-(2-((2-chlorophenyl)amino)-2-oxoethyl)-1*H*-1,2,3-triazol-4-yl)methyl)thio)-5,6-diphenylpyridazine-4-carboxylate (10 h)

Yield (62%, 0.36 g); Dark Yellow solid; m.p. = 161–163 °C; IR (KBr, cm^-1^): 3607, 1730 (C = O), 1639, 1445, 1358, 1166; ^1^ H NMR (DMSO-*d*_6_, 300 MHz): δ = 0.87 (t, *J* = 6.8 Hz, 3 H), 4.03 (q, *J* = 6.7 Hz, 2 H), 4.79 (s, 2 H), 5.44 (s, 2 H), 7.13–7.15 (m, 2 H), 7.28–7.44 (m, 10 H), 7.49 (d, *J* = 7.8 Hz, 1 H), 7.74 (d, *J* = 7.8 Hz, 1 H), 8.16 (s, 1 H), 10.04 (s, 1 H); ^13^ C NMR (DMSO-*d*_6_, 75 MHz): δ 13.2, 24.7, 51.9, 62.0, 125.7, 126.1, 126.6, 127.4, 127.8, 128.2, 128.3, 128.5, 128.76, 128.82, 128.94, 129.5, 129.6, 130.4, 133.6, 134.1, 135.1, 135.9, 155.4, 157.3, 164.0, 164.8; Anal. Calcd. for C_30_H_25_ClN_6_O_3_S: C, 61.59; H, 4.31; N, 14.36. Found: C, 61.80; H, 4.14; N, 14.69; ESI-MS *m/z*: 586.1 [M + 2]^+^.

#### Ethyl 3-(((1-(2-((3-chlorophenyl)amino)-2-oxoethyl)-1*H*-1,2,3-triazol-4-yl)methyl)thio)-5,6-diphenylpyridazine-4-carboxylate (10i)

Yield (60%, 0.35 g); Dark Yellow solid; m.p. = 200–202 °C; IR (KBr, cm^-1^): 3621, 1730 (C = O), 1639, 1429, 1341, 1175; ^1^ H NMR (DMSO-*d*_6_, 300 MHz): δ = 0.87 (t, *J* = 7.0 Hz, 3 H), 4.03 (q, *J* = 6.7 Hz, 2 H), 4.79 (s, 2 H), 5.33 (s, 2 H), 7.12–7.14 (m, 3 H), 7.26–7.44 (m, 10 H), 7.44 (d, *J* = 8.1 Hz, 1 H), 8.13 (s, 1 H), 10.64 (s, 1 H); ^13^ C NMR (DMSO-*d*_6_, 75 MHz): δ 13.3, 24.7, 52.2, 62.0, 117.6, 118.7, 123.4, 125.5, 127.8, 128.2, 128.3, 128.8, 128.9, 129.6, 130.4, 130.5, 133.1, 133.5, 133.6, 135.1, 135.9, 139.8, 155.4, 157.3, 164.0, 164.6; Anal. Calcd. for C_30_H_25_ClN_6_O_3_S: C, 61.59; H, 4.31; N, 14.36. Found: C, 61.31; H, 4.53; N, 14.02; ESI-MS *m/z*: 586.2 [M + 2]^+^.

#### Ethyl 3-(((1-(2-((4-chlorophenyl)amino)-2-oxoethyl)-1*H*-1,2,3-triazol-4-yl)methyl)thio)-5,6-diphenylpyridazine-4-carboxylate (10j)

Yield (74%, 0.43 g); Dark Yellow solid; m.p. = 195–197 °C; IR (KBr, cm^-1^): 3622, 1710 (C = O), 1646, 1442, 1331, 1192; ^1^ H NMR (DMSO-*d*_6_, 500 MHz): δ = 0.89 (t, *J* = 7.0 Hz, 3 H), 4.06 (q, *J* = 6.9 Hz, 2 H), 4.81 (s, 2 H), 5.43 (s, 2 H), 7.13–7.15 (m, 2 H), 7.20–7.30 (m, 10 H), 7.83 (d, *J* = 8.5 Hz, 2 H), 8.18 (s, 1 H), 11.06 (s, 1 H); ^13^ C NMR (DMSO-*d*_6_, 125 MHz): δ 13.8, 25.2, 52.8, 62.6, 119.5, 125.6, 128.3, 128.79, 128.86, 129.1, 129.3, 129.5, 130.0, 130.1, 130.9, 134.1, 135.7, 136.5, 143.1, 145.0, 155.9, 157.9, 164.5, 165.8; Anal. Calcd. for C_30_H_25_ClN_6_O_3_S: C, 61.59; H, 4.31; N, 14.36. Found: C, 61.25; H, 4.60; N, 14.02.

#### Ethyl 3-(((1-(2-((4-bromophenyl)amino)-2-oxoethyl)-1*H*-1,2,3-triazol-4-yl)methyl)thio)-5,6-diphenylpyridazine-4-carboxylate (10k)

Yield (61%, 0.38 g); Dark Yellow solid; m.p. = 224–226 °C; IR (KBr, cm^-1^): 3632, 1730 (C = O), 1651, 1440, 1352, 1191; ^1^ H NMR (DMSO-*d*_6_, 300 MHz): δ = 0.86 (t, *J* = 6.9 Hz, 3 H), 4.02 (q, *J* = 6.8 Hz, 2 H), 4.80 (s, 2 H), 5.33 (s, 2 H), 7.15–7.16 (m, 2 H), 7.27–7.37 (m, 10 H), 7.54 (d, *J* = 8.9 Hz, 2 H), 8.14 (s, 1 H), 10.60 (s, 1 H); ^13^ C NMR (DMSO-*d*_6_, 75 MHz): δ 13.3, 24.7, 52.2, 62.0, 115.4, 121.1, 127.8, 128.2, 128.3, 128.6, 128.8, 128.9, 129.0, 129.6, 130.4, 131.6, 133.6, 135.2, 135.9, 137.7. 155.4, 157.3, 164.0, 164.3; Anal. Calcd. for C_30_H_25_BrN_6_O_3_S: C, 57.24; H, 4.00; N, 13.35. Found: C, 57.00; H, 4.25; N, 13.60; ESI-MS *m/z*: 630.1 [M + 2]^+^.

#### Ethyl 3-(((1-(2-((3,5-dichlorophenyl)amino)-2-oxoethyl)-1*H*-1,2,3-triazol-4-yl)methyl)thio)-5,6-diphenylpyridazine-4-carboxylate (10 l)

Yield (64%, 0.39 g); Dark Yellow solid; m.p. = 185–187 °C; IR (KBr, cm^-1^): 3616, 1733 (C = O), 1631, 1444, 1330, 1166; ^1^ H NMR (DMSO-*d*_6_, 500 MHz): δ = 0.86 (t, *J* = 7.1 Hz, 3 H), 4.02 (q, *J* = 7.0 Hz, 2 H), 4.81 (s, 2 H), 5.40 (s, 2 H), 7.29–7.37 (m, 10 H), 7.77–7.78 (m, 3 H), 8.17 (s, 1 H), 10.90 (s, 1 H).; ^13^ C NMR (DMSO-*d*_6_, 125 MHz): δ 13.8, 25.2, 52.8, 62.6, 106.1, 119.4, 119.8, 126.0, 128.8, 129.1, 129.3, 129.5, 130.1, 130.9, 133.8, 133.9, 134.1, 135.7, 136.5, 143.0, 155.9, 157.9, 164.5, 165.6.; Anal. Calcd. for C_30_H_24_Cl_2_N_6_O_3_S: C, 58.16; H, 3.90; N, 13.57. Found: C, 57.95; H, 4.22; N, 13.80; ESI-MS *m/z*: 622.1 [M + 4]^+^.

#### Ethyl 3-(((1-(2-((3,4-dichlorophenyl)amino)-2-oxoethyl)-1*H*-1,2,3-triazol-4-yl)methyl)thio)-5,6-diphenylpyridazine-4-carboxylate (10 m)

Yield (78%, 0.48 g); Dark Yellow solid; m.p. = 166–168 °C; IR (KBr, cm^-1^): 3606, 1725 (C = O), 1639, 1441, 1330, 1175; ^1^ H NMR (DMSO-*d*_6_, 500 MHz): δ = 0.86 (t, *J* = 6.4 Hz, 3 H), 4.02 (m, 2 H), 4.80 (s, 2 H), 5.35 (s, 2 H), 7.30–7.42 (m, 12 H), 7.54 (s, 1 H), 8.15 (s, 1 H), 10.77 (s, 1 H); ^13^ C NMR (DMSO-*d*_6_, 125 MHz): δ 13.3, 24.7, 52.2, 62.1, 119.2, 120.4, 125.3, 125.5, 127.8, 128.3, 128.6, 128.8, 129.0, 129.6, 130.4, 130.8, 131.1, 133.5, 133.6, 135.1, 135.9, 138.4, 155.4, 157.3, 164.0, 164.8; Anal. Calcd. for C_30_H_24_Cl_2_N_6_O_3_S: C, 58.16; H, 3.90; N, 13.57. Found: C, 58.42; H, 3.61; N, 13.39; ESI-MS *m/z*: 622.3 [M + 4]^+^.

#### Ethyl 3-(((1-(2-((3-cyanophenyl)amino)-2-oxoethyl)-1*H*-1,2,3-triazol-4-yl)methyl)thio)-5,6-diphenylpyridazine-4-carboxylate (10n)

Yield (70%, 0.40 g); Dark Yellow solid; m.p. = 196–198 °C; IR (KBr, cm^-1^): 3601, 2221, 1724 (C = O), 1644, 1439, 1327, 1180; ^1^ H NMR (DMSO-*d*_6_, 500 MHz): δ = 0.89 (t, *J* = 6.4 Hz, 3 H), 4.06 (m, 2 H), 4.81 (s, 2 H), 5.32 (s, 2 H), 7.16–7.21 (m, 5 H), 7.29–7.33 (m, 6 H), 7.48–7.51 (m, 3 H), 8.15 (s, 1 H), 10.40 (s, 1 H); ^13^ C NMR (DMSO-*d*_6_, 125 MHz): δ 13.8, 24.4, 52.7, 62.6, 119.8, 119.9, 126.0, 127.0, 127.1, 128.3, 128.8, 129.1, 129.3, 129.5, 130.2, 130.9, 134.1 (2 C), 135.7, 136.5, 136.6, 142.9, 144.3, 156.0, 158.0, 164.5, 166.4; Anal. Calcd. for C_31_H_25_N_7_O_3_S: C, 64.68; H, 4.38; N, 17.03. Found: C, 64.99; H, 4.55; N, 16.88; ESI-MS *m/z*: 575.2 [M]^+^.

#### Ethyl 3-(((1-(2-((3-nitrophenyl)amino)-2-oxoethyl)-1*H*-1,2,3-triazol-4-yl)methyl)thio)-5,6-diphenylpyridazine-4-carboxylate (10p)

Yield (81%, 0.48 g); Dark Yellow solid; m.p. = 201–203 °C; IR (KBr, cm^-1^): 3592, 1730 (C = O), 1637, 1549, 1432, 1356, 1189; ^1^ H NMR (DMSO-*d*_6_, 300 MHz): δ = 0.87 (t, *J* = 7.0 Hz, 3 H), 4.06 (d, *J* = 6.8 Hz, 2 H), 4.80 (s, 2 H), 5.39 (s, 2 H), 7.13 (t, *J* = 6.8 Hz, 2 H), 7.32–7.45 (m, 9 H), 7.61 (t, *J* = 7.8 Hz, 1 H), 7.91 (t, *J* = 8.0 Hz, 1 H), 8.17 (s, 1 H), 8.58 (s, 1 H), 10.97 (s, 1 H); ^13^ C NMR (DMSO-*d*_6_, 75 MHz): δ 13.3, 24.7, 52.2, 62.1, 113.4, 118.3, 125.1, 127.8, 128.3, 128.4, 128.6, 128.8, 128.9, 129.0, 129.6, 130.4, 133.5, 133.6, 135.2, 136.0, 139.4, 147.9, 155.4, 157.3, 164.0, 165.1; Anal. Calcd. for C_30_H_25_N_7_O_5_S: C, 60.49; H, 4.23; N, 16.46. Found: C, 60.69; H, 3.99; N, 16.19; ESI-MS *m/z*: 595.2 [M]^+^.

#### Ethyl 3-(((1-(2-((4-nitrophenyl)amino)-2-oxoethyl)-1*H*-1,2,3-triazol-4-yl)methyl)thio)-5,6-diphenylpyridazine-4-carboxylate (10q)

Yield (73%, 0.43 g); Dark Yellow solid; m.p. = 221–223 °C; IR (KBr, cm^-1^): 3610, 1712 (C = O), 1636, 1560, 1432, 1352, 1179; ^1^ H NMR (DMSO-*d*_6_, 500 MHz): δ = 0.89 (t, *J* = 6.4 Hz, 3 H), 4.07 (m, 2 H), 4.81 (s, 2 H), 5.35 (s, 2 H), 7.16 (d, *J* = 8.1 Hz, 2 H), 7.29–7.40 (m, 10 H), 7.16 (d, *J* = 8.2 Hz, 2 H), 8.16 (s, 1 H), 10.61 (s, 1 H); ^13^ C NMR (DMSO-*d*_6_, 125 MHz): δ 13.8, 25.2, 52.7, 62.6, 121.2, 121.3, 126.0, 127.8, 128.3, 128.8, 129.1, 129.2, 129.3, 129.5, 130.1, 130.9, 134.1, 135.7, 136.4, 137.8, 155.9, 157.8, 164.5, 164.9; Anal. Calcd. for C_30_H_25_N_7_O_5_S: C, 60.49; H, 4.23; N, 16.46. Found: C, 60.18; H, 4.09; N, 16.66; ESI-MS *m/z*: 596.1 [M + H]^+^.

#### Ethyl 3-(((1-(2-((3-methyl-4-nitrophenyl)amino)-2-oxoethyl)-1*H*-1,2,3-triazol-4-yl)methyl)thio)-5,6-diphenylpyridazine-4-carboxylate (10r)

Yield (65%, 0.39 g); Dark Yellow solid; m.p. = 212–214 °C; IR (KBr, cm^-1^): 3602, 1722 (C = O), 1641, 1551, 1448, 1352, 1182; ^1^ H NMR (DMSO-*d*_6_, 300 MHz): δ = 0.86 (t, *J* = 6.4 Hz, 3 H), 1.10 (s, 3 H), 4.04 (m, 2 H), 4.80 (s, 2 H), 5.37 (s, 2 H), 7.13 (d, *J* = 6.8 Hz, 2 H), 7.28–7.32 (m, 9 H), 7.40–7.43 (m, 1 H), 8.17 (m, 1 H), 8.33 (s, 1 H), 10.85 (s, 1 H); ^13^ C NMR (DMSO-*d*_6_, 75 MHz): δ 13.3, 19.2, 24.7, 52.2, 62.1, 114.5, 123.7, 127.7, 127.8, 128.27, 128.34, 128.6, 128.8, 128.86, 128.97, 129.6, 130.4, 133.2, 133.6, 135.2, 136.0, 137.2, 148.5, 155.5, 157.3, 164.0, 164.8; Anal. Calcd. for C_31_H_27_N_7_O_5_S: C, 61.07; H, 4.46; N, 16.08. Found: C, 60.75; H, 4.19; N, 16.40; ESI-MS *m/z*: 609.2 [M]^+^.

### Biological studies

#### Rat α‑glucosidase assay

Based on the reported method of Lossow et al., rat small intestine α-glucosidase (EC 3.2.1.20) was prepared. The *in-vitro* activity was determined by the measurement of 4-nitrophenol which was released from *para*-nitrophenyl *α*-D glucopyranoside [44, 45]. The preparation of 200 µL was performed as follows: the enzyme solution (190 µL, 0.15 units/ml), different concentrations of target compounds (1, 10, 20, 50, 100, 500 and 1000 µM (5 µL), potassium phosphate buffer. Final compounds were dissolved in DMSO (not exceed than 5% of final volume) and pre-incubated at 37 °C, *p*-nitrophenyl glucopyranoside and then substrate (5 µL, 3 mM), was added to the enzyme solution and incubated for further one hour at 37 °C. Finally, by using Cytation 3 hybrid microplate reader (BioTek, USA) any change in the absorbance was measured at 405 nm. By using GraphPadprism 6.0 (SanDiego, California, USA) (https://www.graphpad.com/scientificsoftware/prism/) was used to obtain IC_50_ values of tested compounds.

### Kinetic analysis

Modes of enzyme inhibition were determined by recording the effect of various concentrations of the substrates 1, 2.5, 5 or 10 mM pNPG for *α*-glucosidase on Lineweaver–Burk plots using calculated V_max_ and K_m_. For inhibition test of *α*-glucosidase: the concentrations applied for compounds **10k** was 0.1, 1, 5 and 10 nM. Inhibition constants (K_i_) were determined by depicting the secondary plot of K_m_ against various concentrations of inhibitors.

### Docking studies

Docking study of compound 10k was done using Autodock 4.2.1 software. The structure of the targeted protein *α*-glucosidase (PDB: 5NN88) was taken from RCSB data bank [26]. The analysis of docking results were performed by Discovery Studio visualizer 4.5.

## Results and discussions

### Chemistry

**Scheme 1:**
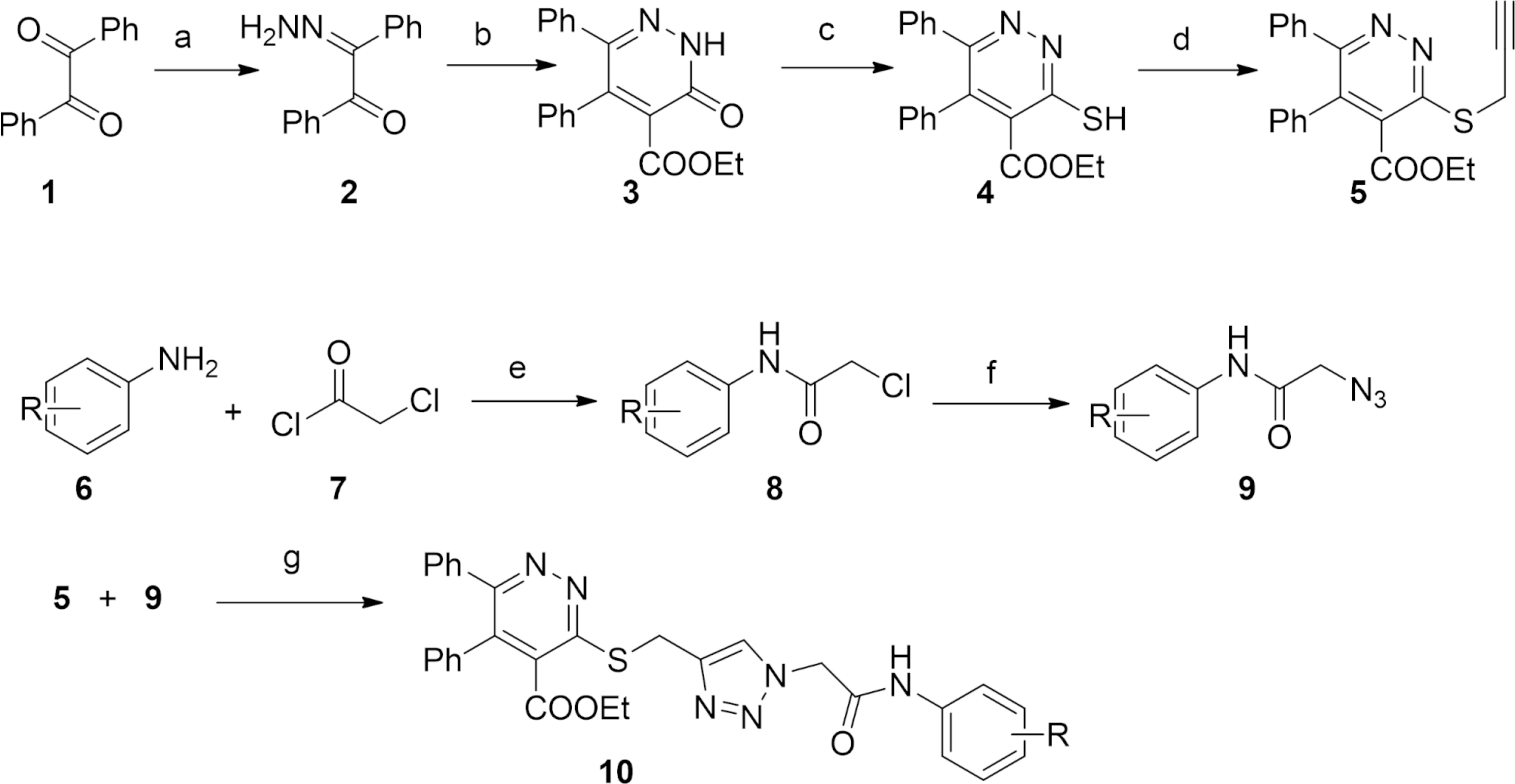
Synthesis of target compounds. Synthesis of target compounds. Reagents and conditions: (a) hydrazine hydrate, methanol, reflux, 15 min.; (b) Na, EtOH, diethyl malonate, reflux; (c) Lawesson’s reagent, toluene, reflux, 18 h; (d) propargyl bromide, DMF, K_2_CO_3_, 50 °C; (e) Et_3_N, DCE, r.t.; (f) NaN_3_, DMSO, r.t.; (g) Et_3_N, DMF, CuI, r.t

The preparation of target compounds is described in scheme 1. Compound **2** was prepared from the previously reported procedure [43, 46]. Subsequently, the cyclization occurred with diethyl malonate and sodium in ethanol at reflux temperature to yield ethyl 3-oxo-5,6-diphenyl-2,3-dihydropyridazine-4-carboxylate. Then, the conversion of carbonyl group into SH was achieved by Lawesson’s reagent in toluene after 18 h heating at reflux condition. The propargylated product **5**, ethyl 5,6-diphenyl-3-(prop-2-yn-1-ylthio)pyridazine-4-carboxylate, was obtained from the reaction of compound **4** and propargyl bromide in dimethyl formamide in the presence of K_2_CO_3_. On the other hand, the reaction of aromatic amines and chloroacetyl chloride was conducted in 1,2-dichloroethane and in the presence of triethylamine as a proton acceptor to obtain 2-chloro-*N*-arylacetamide. After work-up and crystallization, the substitution reaction of compound **8 **with sodium azide in DMSO yielded compound **9**. The click reaction between compound **5** and **9** in DMF and in the presence of triethylamine and catalytic amounts of CuI resulted in target compounds **10a-r** in good yields.

The ^1^H NMR of the most potent compound, **10k**, in DMSO-*d*_6_ was recorded and clearly showed the characteristic triazole and NH protons as singlets at 8.14, 10.60 ppm, respectively. SCH_2_ and NCH_2_ were resonated as singlets at 4.80 and 5.33 ppm, respectively. The ethoxy group was appeared at 0.86 as triplet (*J* = 6.9 Hz) and at 4.02 as quartet (*J* = 6.8 Hz). The aromatic protons near bromine were appeared at 7.54 ppm as doublet showing the *ortho* coupling constant of 8.9 Hz. Besides, other aromatic protons as multiplets at appropriate chemical shifts confirmed the proposed structure. The ^13^C NMR spectrum in the same solvent at 75 MHz showed four aliphatic carbons at 13.3, 24.7, 52.2, 62.0 ppm related to CH_3_, SCH_2_, NCH_2_ OCH_2_. The number of aromatic carbons in the normal range is in accordance to the proposed structure.

### In vitro α-glucosidase inhibitory activities

The SAR studies divulged that different substituents either electron-donating or withdrawing on aromatic moiety along with their positions affected the inhibition. The results are shown as IC_50_s in Table 1. Compounds with unsubstituted phenyl ring and different substituents including methyl, methoxy, isopropyl, fluorine, chlorine, bromine, nitro and cyano at different positions of aryl ring were found to have varying degree of inhibitory potential. Except few ones, all compounds were active against *α*-glucosidase enzyme (IC_50s_ = 1.7 to 86.5 µM). Compound **10k** bearing 4-bromo substituent was found to be 100 folds more active with IC_50_ value of 1.7 µM compared to standard drug (IC_50_ = 173 µM). Compound 10l having 3,5-*di*chloro group was the second most active compound. Further studies indicated that compound **10 h** bearing chlorine (–Cl) group at *ortho* position of phenyl ring displayed stronger *α*-glucosidase inhibitory activity (IC_50_ = 14.9 µM) than *meta*- and *para*-chloro substituted analogues (**10i** and **10j**). Interestingly, *p*-bromo substituted analogue (**10k**) showed strong inhibitory activity compared to *p*-fluoro and *p*-chloro analogues. Interesting to observe that compound **10i** exhibited no inhibitory activity, while the combination of second chlorine atom (–Cl) generated active compounds (10 l, **10 m**). Electron-releasing substituted analogues is more active than electron-withdrawing substituted compounds which are exemplified by *meta*-methoxy, methyl (*m*–OMe, *m*-Me) substituted 1,2,3-triazoles being more active than *meta*-nitro (*m*–NO_2_) and *meta*-cyano (*m*–CN) substituted analogues. While, 4-nitro substituted compound **10q** (IC_50_ = 47.1 µM) was active, the introduction of methyl at *meta* position resulted in no activity at the resultant compound, **10r**. The movement of electron-withdrawing groups meaning nitro and cyano from *meta* to *para* generated active compounds (**10o **and **10q**). This trend was also observed in the case of chlorine-substituted compounds (**10i***vs*. **10j**). The cytotoxicity of compound **10k**, the most active compound, was investigated against the normal cell line. No toxicity was observed against HDF [28].

**Table 1:** *In vitro α*-glucosidase inhibitory activities and yields of target compounds.^a,b^

Entry	Compound	R	IC_50_ (µΜ)	Yield (%)^c^	
**1**	**10a**	H	35.6 ± 8.9	65	
**2**	**10b**	3-Me	58.9 ± 7.5	71	
**3**	**10c**	4-Me	> 250	70	
**4**	**10d**	4-*i*Pr	86.5 ± 7.3	68	
**5**	**10e**	3-OMe	35.7 ± 3.9	72	
**6**	**10f**	4-OMe	25.2 ± 2.5	66	
**7**	**10 g**	4-F	37.5 ± 7.2	79	
**8**	**10 h**	2-Cl	14.9 ± 1.9	62	
**9**	**10i**	3-Cl	> 250	60	
**10**	**10j**	4-Cl	21.0 ± 5.3	74	
**11**	**10k**	4-Br	1.7 ± 0.12	61	
**12**	**10 L**	3,5-*di*Cl	14.1 ± 3.3	64	
**13**	**10 m**	3,4-*di*Cl	27.7 ± 3.0	78	
**14**	**10n**	3-CN	> 250	70	
**15**	**10o**	4-CN	28.9 ± 3.3	77	
**16**	**10p**	3-NO_2_	84.0 ± 13.2	81	
**17**	**10q**	4-NO_2_	47.1 ± 5.6	73	
**18**	**10r**	3-Me-4-NO_2_	> 250	65	
**19**	Acarbose		170.5 ± 23.1		

^a^ Values are the means of three replicates ± standard deviation

^b^ The activity against rat small intestine *α*-glucosidase

^c^ Isolated yields

### Kinetic studies

**Fig. 2:**
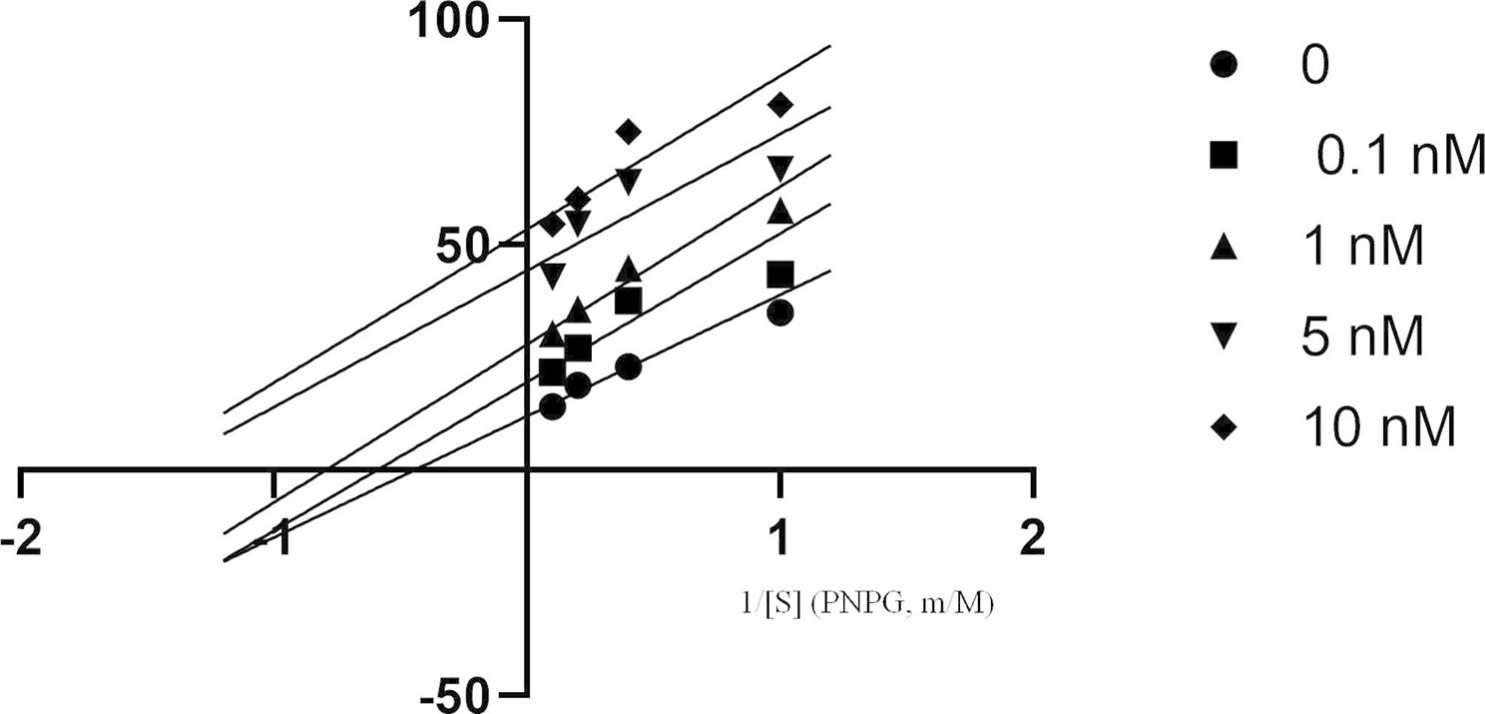
The relative activity against the concentrations of *α*-glucosidase in the presence of compound **10k** (0, 0.1, 1, 5 and 10 nM)

The activity of 4-nitrophenyl-*β*-*D*-galactopyranoside (PNPG) was investigated in the presence and absence of compound **10k**. The results were analyzed by Lineweaver-Burk and confirmed that this compound induced uncompetitive inhibition on *α*-glucosidase (Fig. 2). The slope was not altered by the presence of this compound and increasing concentrations of the substrate resulted in parallel lines. By using secondary plot, the inhibitor constant K_i_ was determined to be 125 nM.

### Docking studies

**Fig. 3:**
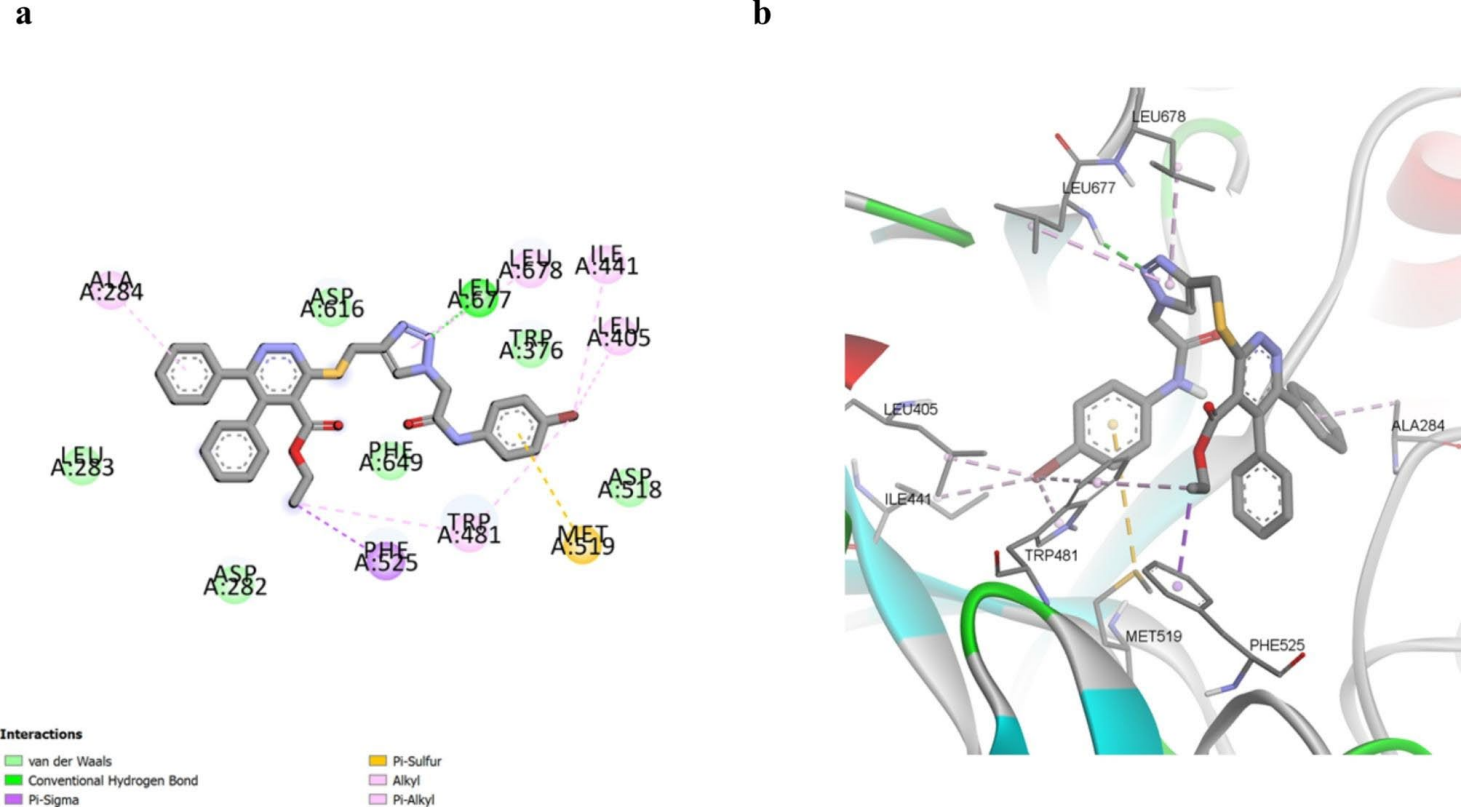
2D (a) and 3D (b) binding modes the most active **10k** skeleton

To gain insights into possible binding modes with *α*-glucosidase, molecular docking was performed and the results are shown in Fig. 3. It is observed that the hydrogen bond, pi-sigma and the pi-sulfur and pi-alkyl interactions are the main interactions of compound **10k** contributing to the binding affinity of this compound with the enzyme. The most potent compound exhibited favorable interactions with amino acid residues. The triazole ring formed hydrogen bond with Leu677 to enhance the binding affinity. The van der Waals interactions are formed with Phe649, Trp 376, Asp616, Leu283, Asp282. The ethoxy group could form pi-sigma interaction with residue of the enzyme.

## Conclusion

In summary, a newly synthesized series of pyridazine-triazole derivatives were synthesized from benzil and their inhibitory activity toward *α*-glucosidase were also evaluated. All synthesized compounds were new and evaluated by IR, ^1^H NMR, ^13^C NMR and Mass spectroscopy. The obtained results showed that most of the target compounds showed significant inhibitory potency. The kinetic studies revealed that the inhibition was uncompetitive. Besides, the suitable interactions and no toxicity of the most potent compound confirmed that these derivatives could be regarded as a good candidate for further investigation and optimization.

## Electronic supplementary material

Below is the link to the electronic supplementary material.

Supplementary Material 1

## Data Availability

All data generated or analyzed during this study are included in this published article and its additional information files.

## Declarations

### Supporting information

Copies of ^1^ H-NMR ^13^ C-NMR spectra are available as supplementary data.

## References

[1] Al-Hassan N (2003). Definition of diabetes mellitus. Br J Gen Pract.

[2] World Health Organization., Global report on diabetes. https://www.who.int/health-topics/diabetes/en/ (assessed 24 May 2022)

[3] Kannel WB, Mcgee DL (1979). Diabetes and cardiovascular risk factors: the Framingham study. Circulation.

[4] Krolewski AS, Kosinski EJ, Warram JH, Leland OS, Busick EJ, Asmal AC, Rand LI, Christlieb AR, Bradley RF, Kahn CR (1987). Magnitude and determinants of coronary artery disease in juvenile-onset, insulin-dependent diabetes mellitus. Am J Cardiol.

[5] Ogurtsova K, Rocha Fernandes JD, Huang Y, Linnenkamp U, Guariguata L, Cho NH, Cavan D, Shaw JE, Makaroff LE (2017). IDF Diabetes Atlas: global estimates for the prevalence of diabetes for 2015 and 2040. Diabetes Res Clin Pract.

[6] Gloster TM, Davies GJ (2010). Glycosidase inhibition: assessing mimicry of the transition state. Org Biomol Chem.

[7] Liu Z, Ma S (2017). Recent advances in synthetic α-Glucosidase inhibitors. Chem Med Chem.

[8] Hossain MA, Pervin R. Current antidiabetic drugs: review of their efficacy and safety in: nutritional and therapeutic interventions for diabetes and metabolic. Elsevier; 2018. pp. 455–73.

[9] He ZX, Zhou ZW, Yang Y, Yang T, Pan SY, Qiu JX, Zhou SF (2015). Overview of clinically approved oral antidiabetic agents for the treatment of type 2 diabetes mellitus. Clin Exp Pharmacol Physiol.

[10] Yee HS, Fong NT (1996). A review of the Safety and Efficacy of Acarbose in Diabetes Mellitus. Pharmacotherapy.

[11] Kaku K (2014). Efficacy of voglibose in type 2 diabetes. Pharmacotherapy.

[12] Scott LJ, Spencer CM (2000). Miglitol: a review of its therapeutic potential in type 2 diabetes Mellitus. Drugs.

[13] Dhameja M, Gupta P (2019). Synthetic heterocyclic candidates as promising α-Glucosidase inhibitors: an overview. Eur J Med Chem.

[14] Ali M, Khan KM, Mahdavi M, Jabbar A, Shamim S, Salar U, Taha M, Perveen S, Larijani B, Faramarzi MA (2020). Synthesis, in vitro and in silico screening of 2-amino-4-aryl-6-(phenylthio) pyridine-3,5-dicarbonitriles as novel α-glucosidase inhibitors. Bioorg Chem.

[15] Hameed S, Khan KM, Taslimi P, Salar U, Taskin-Tok T, Kisa D, Saleem F, Solangi M, Ahmad MHU, Kiran Rani A (2022). Evaluation of synthetic 2-aryl quinoxaline derivatives as α-amylase, α-glucosidase, acetylcholinesterase, and butyrylcholinesterase inhibitors. Int J Biol Macromol.

[16] Bushra SS, Khan KM, Ullah N, Mahdavi M, Faramarzi MA, Larijani B, Salar U, Rafique R, Taha M, Perveen S (2021). Synthesis, in vitro, and in silico evaluation of Indazole Schiff bases as potential α-glucosidase inhibitors. J Mol Struct.

[17] Yousuf H, Shamim S, Khan KM, Chigurupati S, Kanwal, Hameed S, Khan MN, Taha M, Arfeen M (2020). Dihydropyridines as potential α-amylase and α-glucosidase inhibitors: synthesis, in vitro and in silico studies. Bioorg Chem.

[18] Bansal R, Thota S (2013). Pyridazin-3(2H)-ones: the versatile pharmacophore of medicinal significance. Med Chem Res.

[19] Loksha YM, Abd-Alhasee MM. Synthesis and biological screening of some novel 6-substituted 2-alkylpyridazin-3(2H)-ones as anti-inflammatory and analgesic agents. Arch Pharm Chem Life Sci. 2020;1900295. 10.1002/ardp.201900295 31944384

[20] Singh B, Bhatia R, Pani B, Gupta D (2020). Synthesis, crystal structures and biological evaluation of new pyridazine derivatives. J Mol Struct.

[21] Harris RR, Black L, Surapaneni S, Kolasa T, Majest S, Namovic MT, Grayson G, Komater V, Wilcox D, King L, Marsh K, Jarvis MF, Nuss M, Nellans HL, Pruesser GA, Reinhart B, Cox P, Jacobson A, Stewart M, Carter CJ (2004). ABT-963 [2-(3,4-difluoro-phenyl)-4-(3-hydroxy-3-methyl-butoxy)-5-(4-methanesulfonyl-phenyl)-2H-pyridazin-3-one], a highly potent and selective disubstituted pyridazinone cyclooxgenase-2 inhibitor. J Pharmacol Exp Ther.

[22] Sharma D, Bansal R (2016). Synthesis of 2-substituted-4-aryl-6-phenylpyridazin-3(2H)- ones as potential anti-inflammatory and analgesic agents with cardioprotective and ulcerogenic sparing effects. Med Chem Res.

[23] Krall J, Bavo F, Falk-Petersen CB, Jensen CH, Nielsen JO, Tian Y, Anglani V, Kongstad KT, Piilgaard L, Nielsen B, Gloriam DE, Kehler J, Jensen AAK, Harpsøe K, Wellendorph P, Frølund B (2019). Discovery of 2-(Imidazo[1,2-b]pyridazin 2-yl)acetic acid as a New Class of Ligands Selective for the γ-Hydroxybutyric AcidGHB) High-Affinity binding Sites. J Med Chem.

[24] Sergeev PG, Nenajdenko VG (2020). Recent advances in the chemistry of pyridazine an important representative of six-membered nitrogen heterocycles. Russ Chem Rev.

[25] Ahmed EM, Hassan MSA, El-Malah AA, Kassab AE (2020). New pyridazine derivatives as selective COX-2 inhibitors and potential antiinflammatory agents; design, synthesis and biological evaluation. Bioorg Chem.

[26] Deora GS, Qin CX, Vecchio EA, Debono AJ, Priebbenow DL, Brady RM, Beveridge J, Teguh SC, Deo M, May LT, Krippner G, Ritchie RH, Baell JB (2019). Substituted Pyridazin-3(2H)-ones as highly potent and biased formyl peptide receptor agonists. J Med Chem.

[27] Kolb HC, Finn MG, Sharpless KB (2001). Click Chemistry: diverse chemical function from a few good reactions. Angew Chem Int Ed.

[28] Wu P, Feldman AK, Nugent K, Hawker CJ, Scheel A, Voit B, Pyun J, Frechet JMJ, Sharpless KB, Fokin V (2004). Efficiency and fidelity in a click-chemistry route to triazole dendrimers by the copper(i)-catalyzed ligation of azides and alkynes. Angew Chem Int Ed.

[29] Tron GC, Pirali T, Billington RA, Canonico PL, Sorba G, Genazzani A (2008). Click chemistry reactions in medicinal chemistry: applications of the 1,3-dipolar cycloaddition between azides and alkynes. Med Res Rev.

[30] Rani A, Singh G, Singh A, Maqbool U, Kaur G, Singh J (2010). CuAAC-Ensembled 1,2,3-TriazoleLinked Isosteres as Pharmacophores in Drug Discovery. RSC Adv.

[31] Jiang X, Hao X, Jing L, Wu G, Kang D, Liu X, Zhan P (2019). Recent applications of click Chemistry in Drug Discovery. Expert Opin Drug Disc.

[32] Moghimi S, Salarinejad S, Toolabi M, Firoozpour L, Sadat Ebrahimi SE, Safari F, Madani-Qamsari F, Mojtabavi S, Faramarzi MA, Karima S, Pakrad R, Foroumadi A (2021). Synthesis, in-vitro evaluation, molecular docking, and kinetic studies of pyridazine-triazole hybrid system as novel α-glucosidase inhibitors. Bioorg Chem.

[33] Peytam F, Takalloobanafshi G, Saadattalab T, Norouzbahari M, Emamgholipour Z, Moghimi S, Firoozpour L, Bijanzadeh HR, Faramarzi MA, Mojtabavi S, Rashidi–Ranjbar P, Karima S, Pakraad R, Foroumadi A (2021). Design, synthesis, molecular docking, and in vitro α–glucosidase inhibitory activities of novel 3-amino–2,4–diarylbenzo[4,5]imidazo[1,2–a]pyrimidines against yeast and rat α–glucosidase. Sci Rep.

[34] Peytam F, Adib M, Shourgeshty R, Firoozpour L, Rahmanian-Jazi M, Jahani M, Moghimi S, Divsalar K, Faramarzi MA, Mojtabavi S, Safari F, Mahdavi M, Foroumadi A (2020). An efficient and targeted synthetic approach towards new highly substituted 6-aminopyrazolo[1,5-a] pyrimidines with α-glucosidase inhibitory activity. Sci Rep.

[35] Moghimi S, Toolabi M, Salarinejad S, Firoozpour L, Sadat Ebrahimi SE, Safari F, Mojtabavi S, Faramarzi MA, Foroumadi A (2020). Design and synthesis of novel pyridazine N-aryl acetamides: In-vitro evaluation of α-glucosidase inhibition,docking, and kinetic studies. Bioorg Chem.

[36] Wang J, Wang D, He X, Li J, Li Z, Peng G (2016). Synthesis and biological evaluation of novel 1,2,4-triazine derivatives bearing carbazole moiety as potent α-glucosidase inhibitors. Bioorg Med Chem Lett.

[37] Wang G, Peng Z, Wang J, Li X, Li J (2017). Synthesis, in vitro evaluation and molecular docking studies of novel triazine-triazole derivatives as potential a-glucosidase inhibitors. Eur J Med Chem.

[38] Wang G, Li X, Wang J, Xie Z, Li L, Chen M, Chen S, Peng Y (2017). Synthesis, molecular docking and α-glucosidase inhibition of 2-((5,6-diphenyl-1,2,4-triazin-3-yl)thio)-N-arylacetamides. Bioorg Med Chem Lett.

[39] Wang G, Peng Z, Gong Z, Li Y (2018). Synthesis, biological evaluation, and docking studies of novel 5,6-diaryl-1,2,4-triazine thiazole derivatives as a new class of α-glucosidase inhibitors. Bioorg Chem.

[40] Shamim S, Khan KM, Ullah N, Chigurupati S, Wadood A, Rehman AU, Ali M, Salar U, Alhowail A, Taha M, Perveen S (2020). Synthesis and screening of (*E*)-3-(2-benzylidenehydrazinyl)-5,6-diphenyl-1,2,4-triazine analogs as novel dual inhibitors of α-amylase and α-glucosidase. Bioorg Chem.

[41] Ali F, Khan KM, Salar U, Taha M, Ismail NH, Wadood A, Riaz M, Perveen S (2017). Hydrazinyl arylthiazole based pyridine scaffolds: synthesis, structural characterization, in vitro α-glucosidase inhibitory activity, and in silico studies. Eur J Med Chem.

[42] Basha FZ (2017). New carbazole linked 1,2,3-triazoles as highly potent non-sugar α-glucosidase inhibitors. Bioorg Chem.

[43] Al-kahraman MSAY, Al-kahraman YM, Singh GS (2012). Evaluation of some classical hydrazones of ketones and 1,2-diketones as antileishmanial, antibacterial and antifungal agents. Arch Pharm Res.

[44] Lossow WJ, Migliorini RH, Brot N, Chaikoff IL (1964). Effect of total exclusion of the exocrine pancreas in the rat upon in vitro esterification of C14 – labeled cholesterol by the intestine and upon lymphatic absorption of C14 – labeled cholesterol. J Lipid Res.

[45] Kim JH, Cho CW, Kim HY, Kim KT, Choi GS, Kim HH, Cho IS, Kwon SJ, Choi SK, Yoon JY, Yang SY, Kang JS (2017). KimYH. α-Glycosidase inhibition by prenylated and lavandulyl compounds from Sophora flavescens roots and in silico analysis. Int J Biol Macromol.

[46] Schmidt P, Druey J (1954). Heilmittelchemische Studien in der heterocyclischen Reihe. Mitteilung. Pyridazine II. Eine neue Pyridazinsynthese. Helv Chim Acta.

