# Supplementary material for: Synthesis, α-Glucosidase inhibitory activity and docking studies of Novel Ethyl 1,2,3-triazol-4-ylmethylthio-5,6-diphenylpyridazine-4-carboxylate derivatives
Source: BMC Chem. 2023 Jun 26;17(1):66. doi: 10.1186/s13065-023-00973-8 (PMC10294378; doi:10.1186/s13065-023-00973-8)

**Synthesis, α-Glucosidase Inhibitory Activity and Docking Studies of Novel Ethyl 1,2,3-triazol-4-ylmethylthio-5,6-diphenylpyridazine-4-carboxylate Derivatives**

Loghman Firoozpour^a^, Setareh Moghimi^b^, Somayeh Salarinejad^a^, Mahsa Toolabi^c^, Mahdi Rafsanjani^a^, Roya Pakrad^d^, Farzaneh Salmani^d^, Seyed Mohammad Shokrolahi^d^, Seyed Esmail Sadat Ebrahimi^a^, Saeed Karima^d^, Alireza Foroumadi^a,b*^

^a^ Department of Medicinal Chemistry, Faculty of Pharmacy, Tehran University of Medical Sciences, Tehran, Iran

^b^ Drug Design and Development Research Center, The Institute of Pharmaceutical Sciences (TIPS), Tehran University of Medical Sciences, Tehran, Iran

^c^ Department of Medicinal Chemistry, School of Pharmacy, Ahvaz Jundishapur University of Medical Sciences, Ahvaz, Iran

^d^ Department of Clinical Biochemistry, School of Medicine, Shahid Beheshti University of Medical Sciences (SBMU), Tehran, Iran

^*^ Corresponding author. Tel: +98-21-66954708; Fax: +98-21-66461178; e-mail: [aforoumadi@yahoo.com](mailto:aforoumadi@yahoo.com)

^1^H NMR spectrum of compound 10a


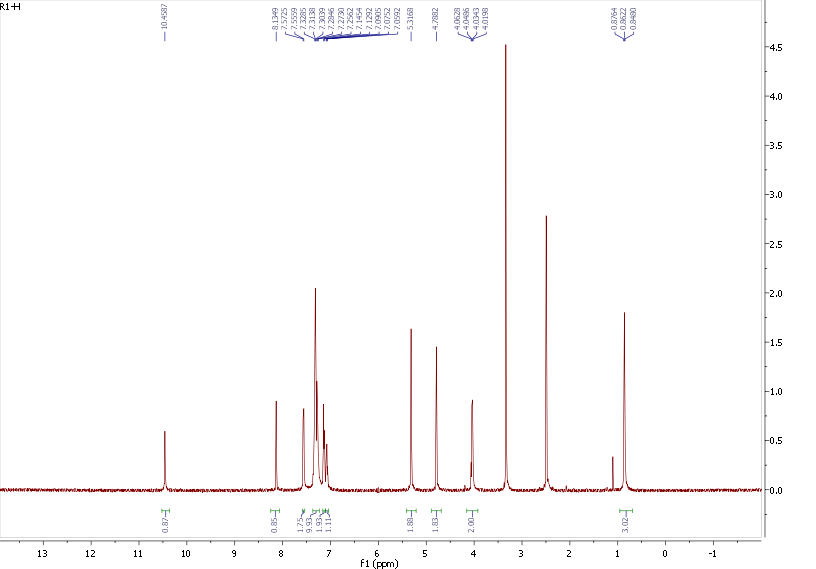


^1^H NMR spectrum of compound 10b


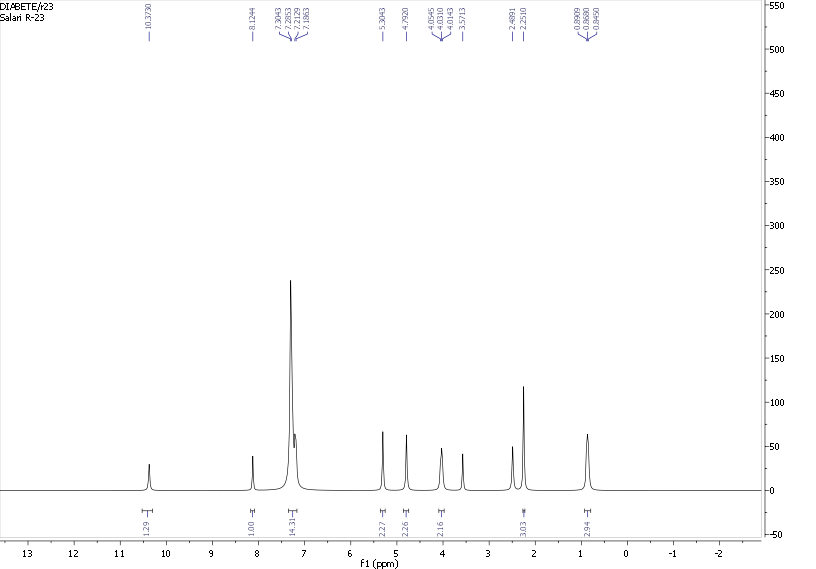


^13^C NMR spectrum of compound 10b


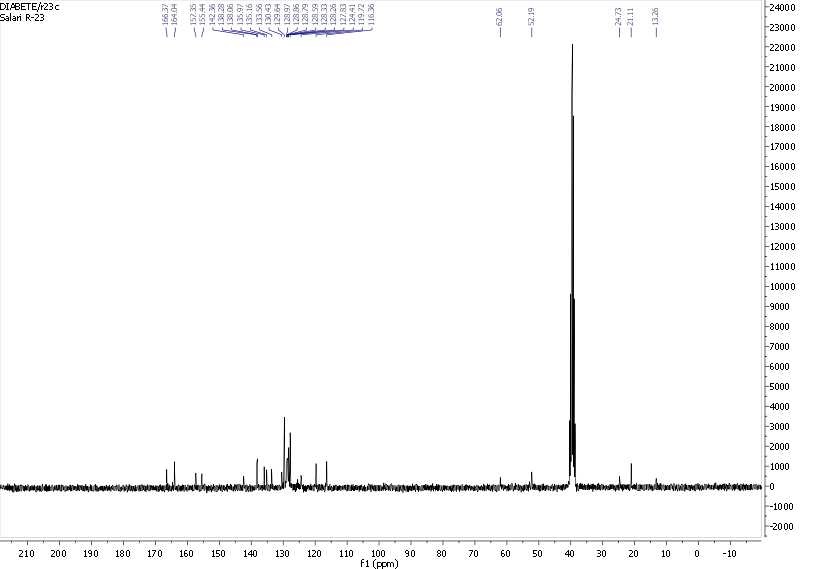


^1^H NMR spectrum of compound 10c


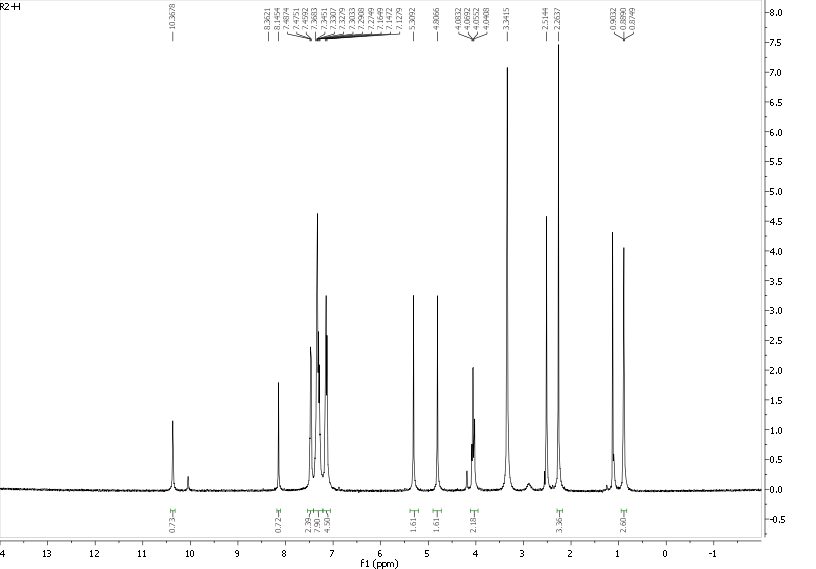


^1^H NMR spectrum of compound 10f


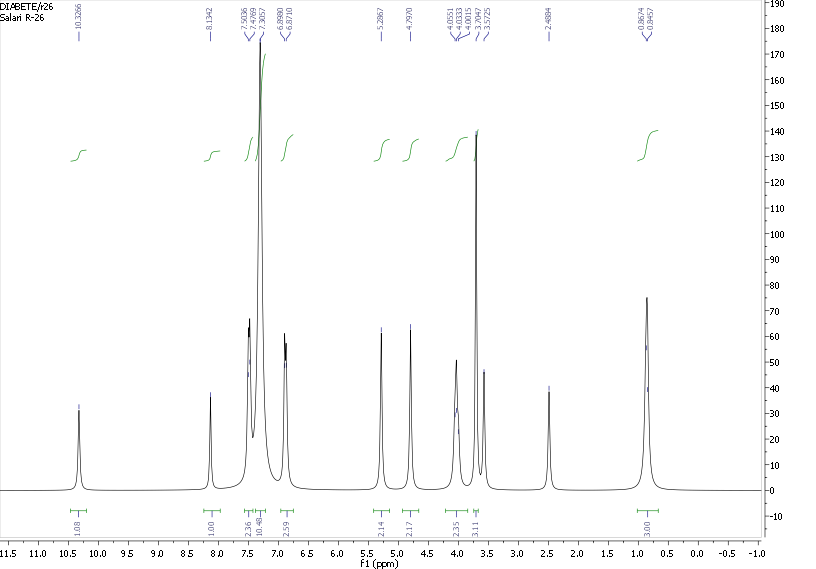


^13^C NMR spectrum of compound 10f


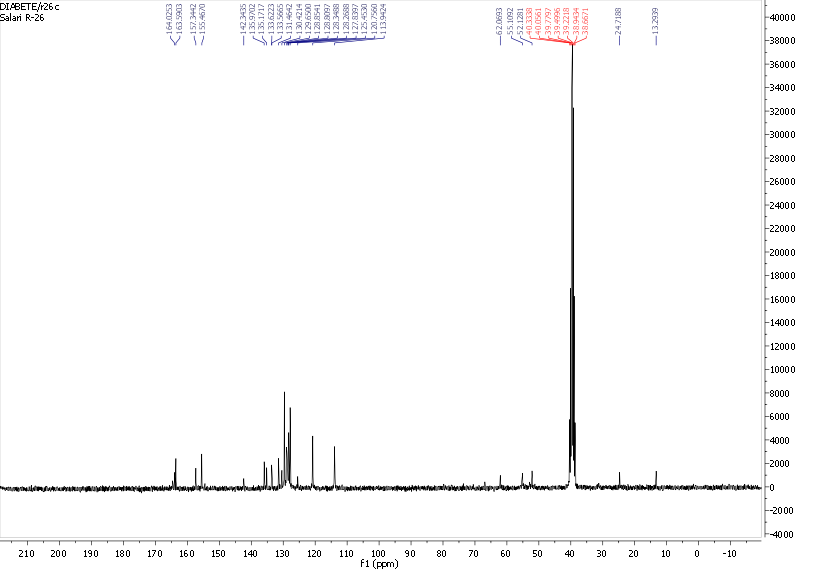


^1^H NMR spectrum of compound 10h


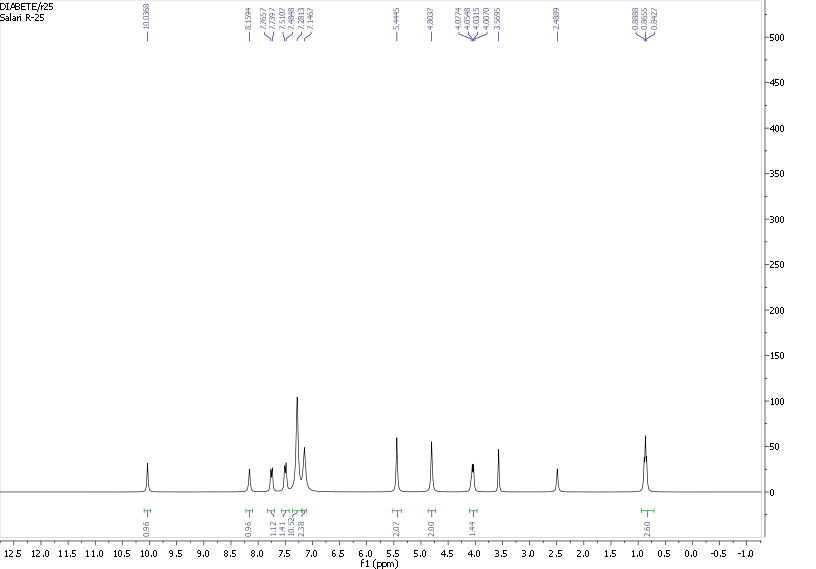


^13^C NMR spectrum of compound 10h


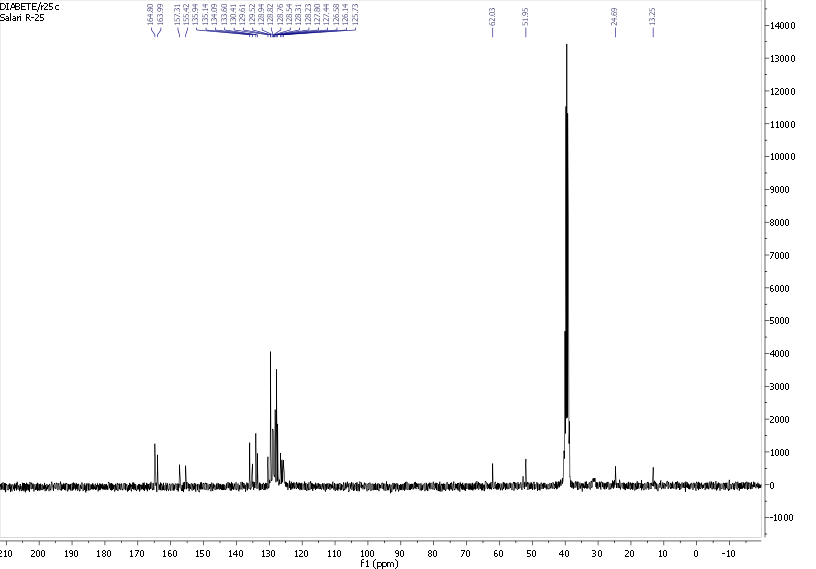


^1^H NMR spectrum of compound 10i


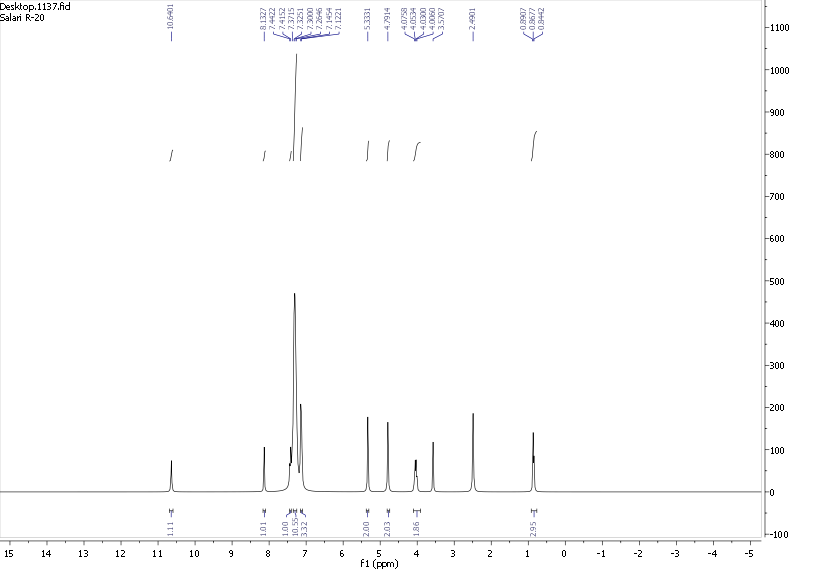


^13^C NMR spectrum of compound 10i


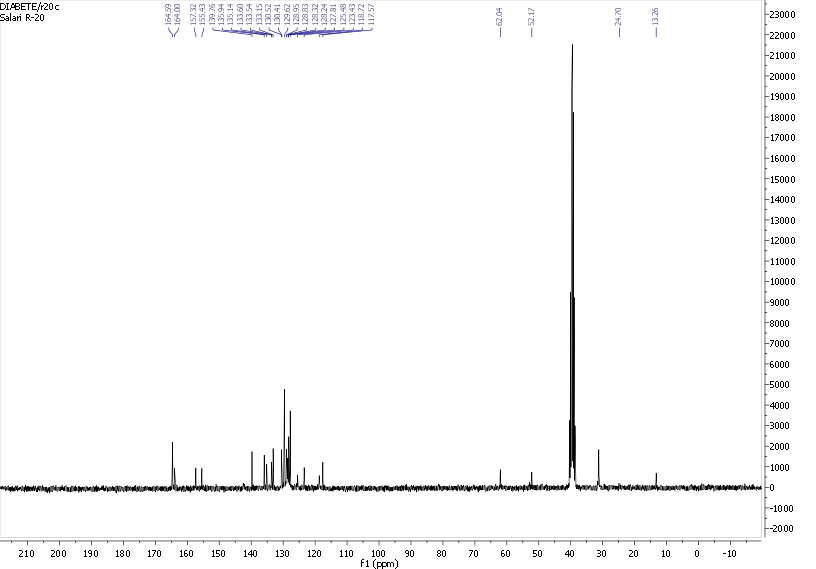


^1^H NMR spectrum of compound 10j


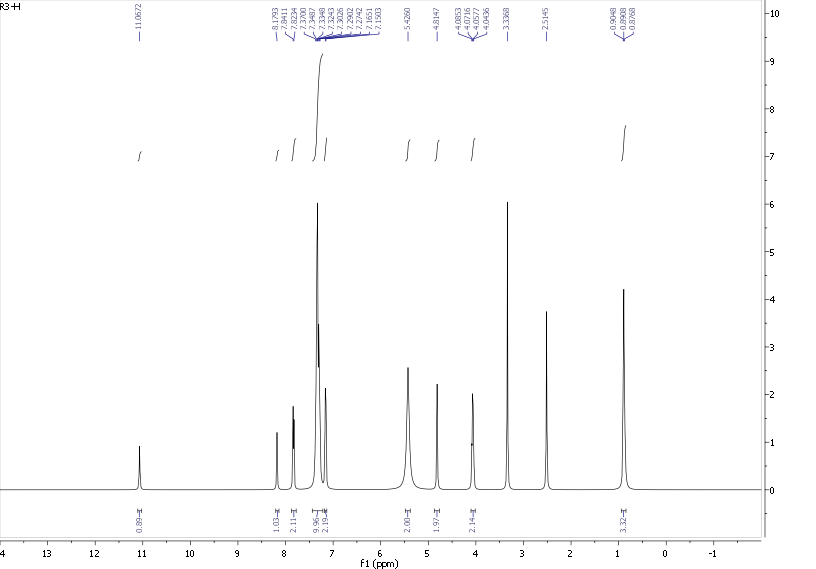


^13^C NMR spectrum of compound 10j


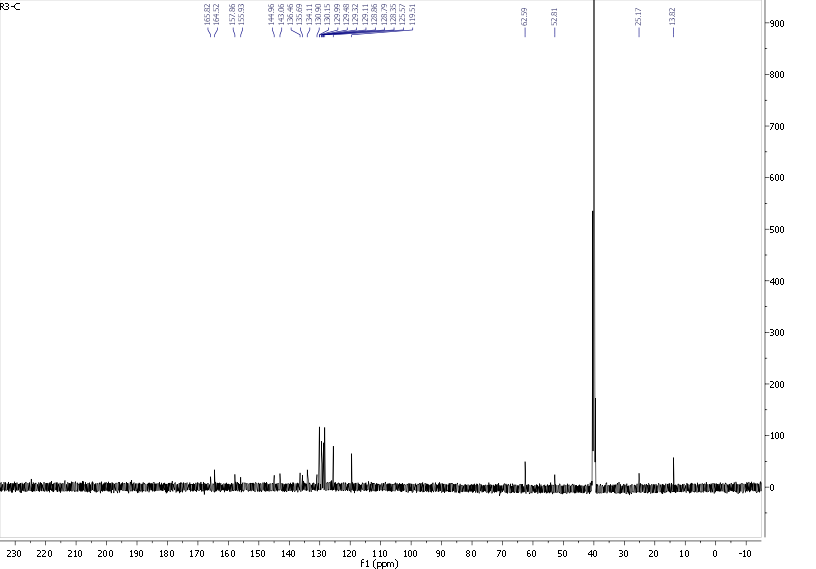


^1^H NMR spectrum of compound 10k


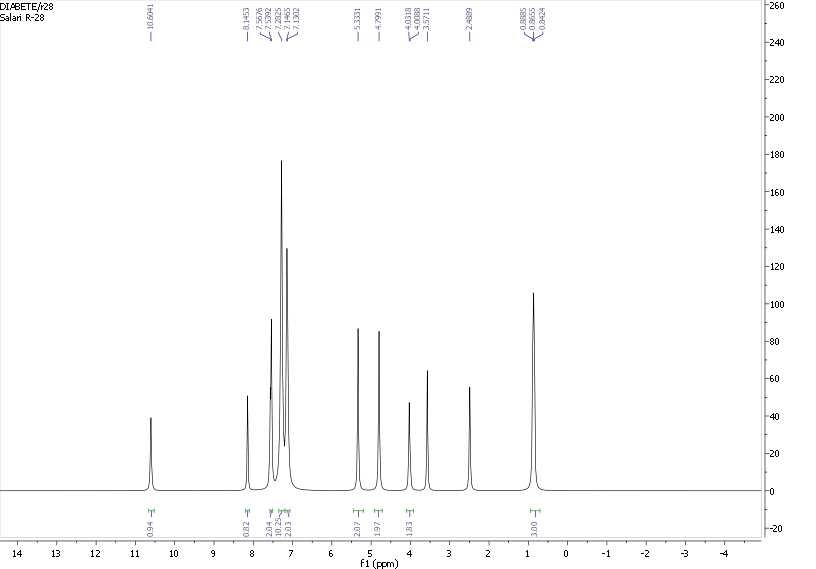


^13^C NMR spectrum of compound 10k


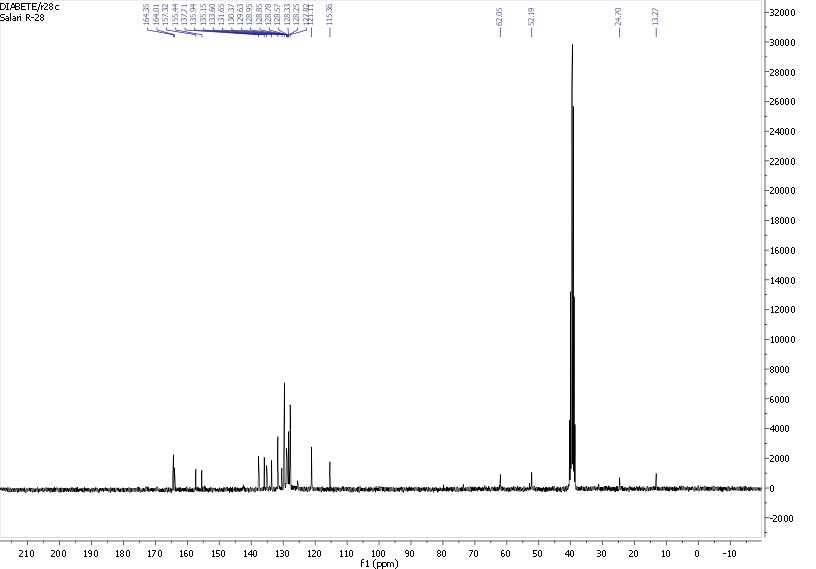


^1^H NMR spectrum of compound 10l


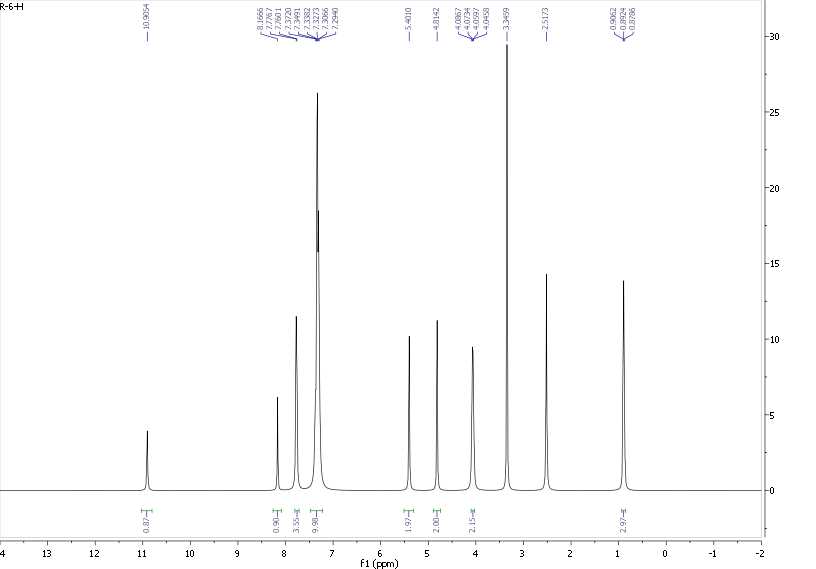


^13^C NMR spectrum of compound 10l


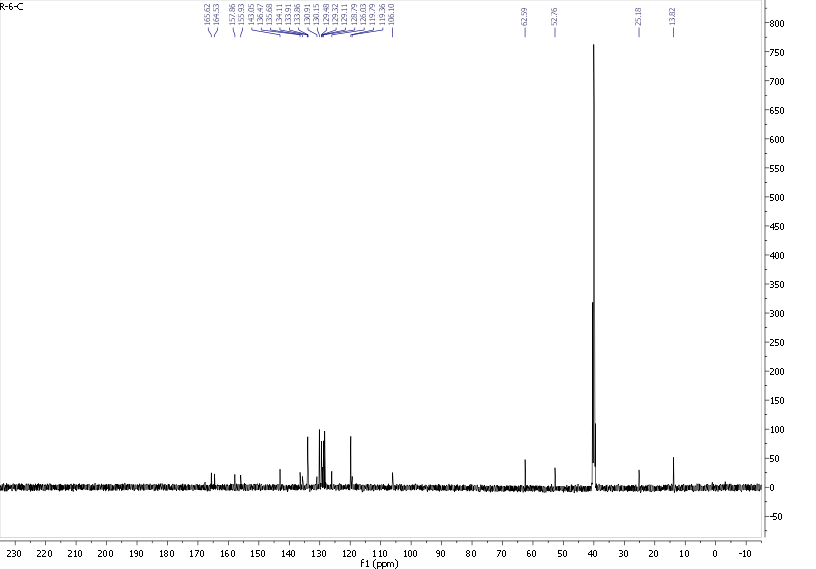


^1^H NMR spectrum of compound 10m
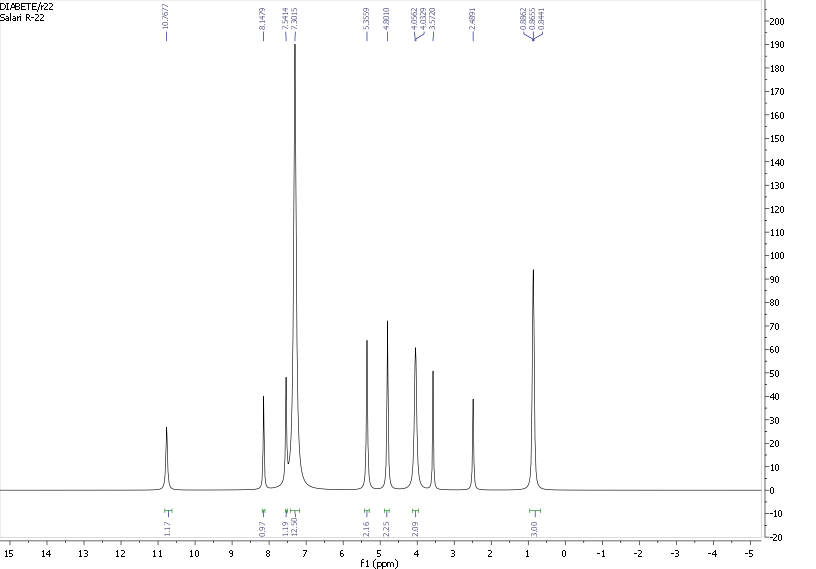


^13^C NMR spectrum of compound 10m


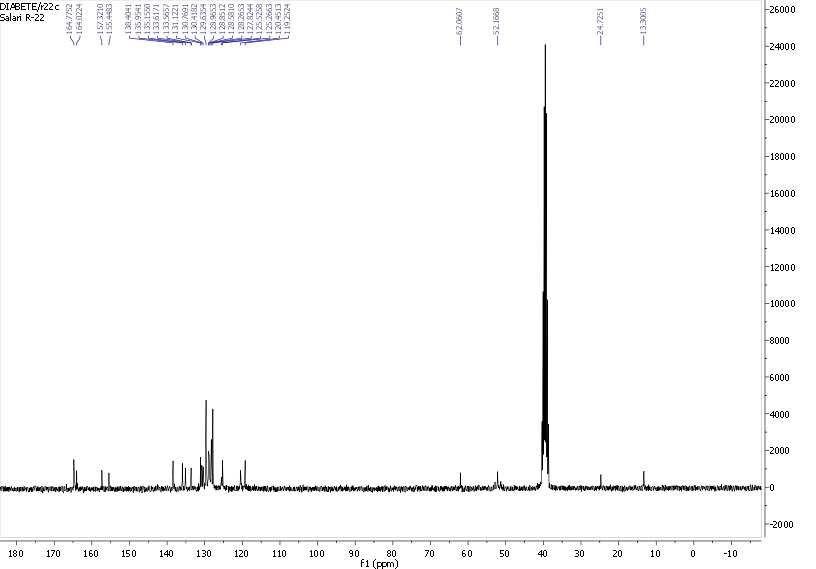


^1^H NMR spectrum of compound 10n


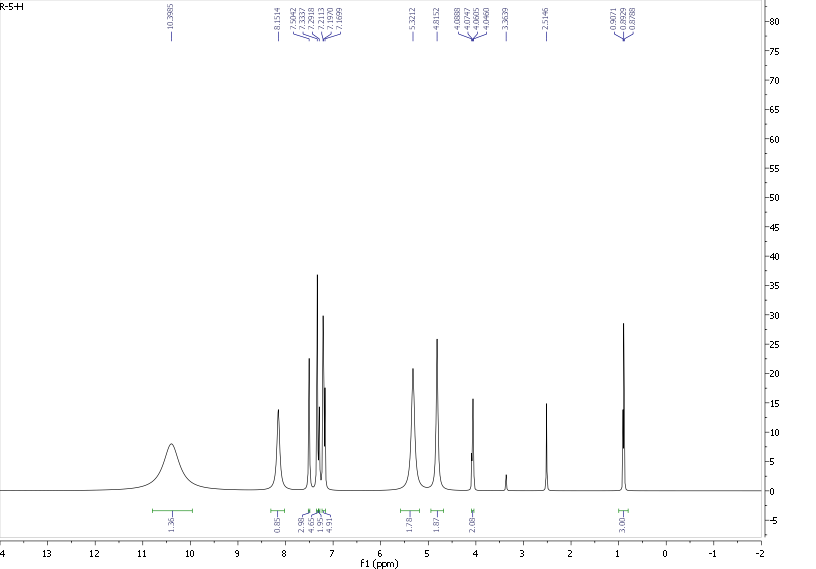


^13^C NMR spectrum of compound 10n


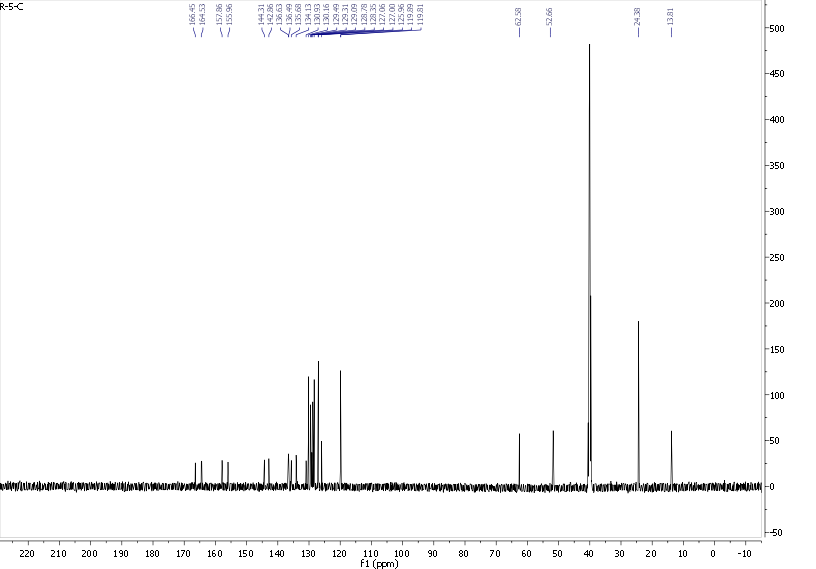


^1^H NMR spectrum of compound 10p


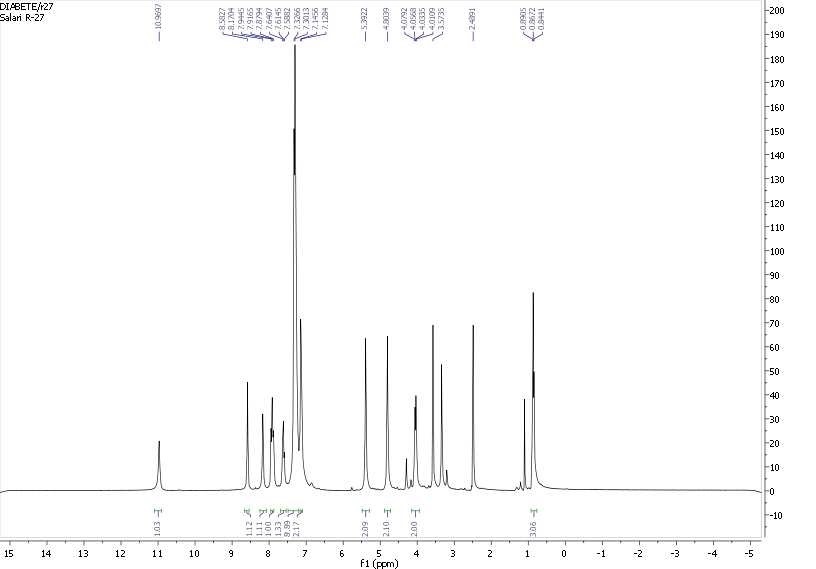


^13^C NMR spectrum of compound 10p


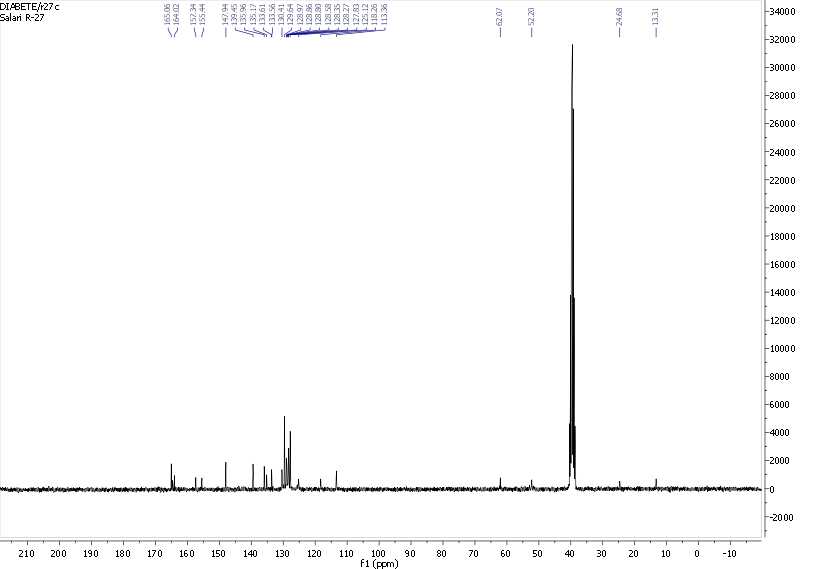


^1^H NMR spectrum of compound 10q


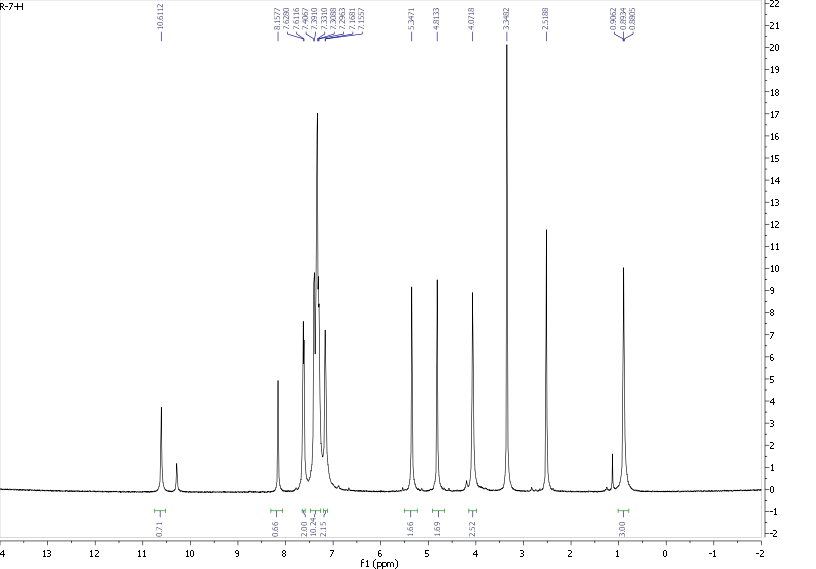


^13^C NMR spectrum of compound 10q


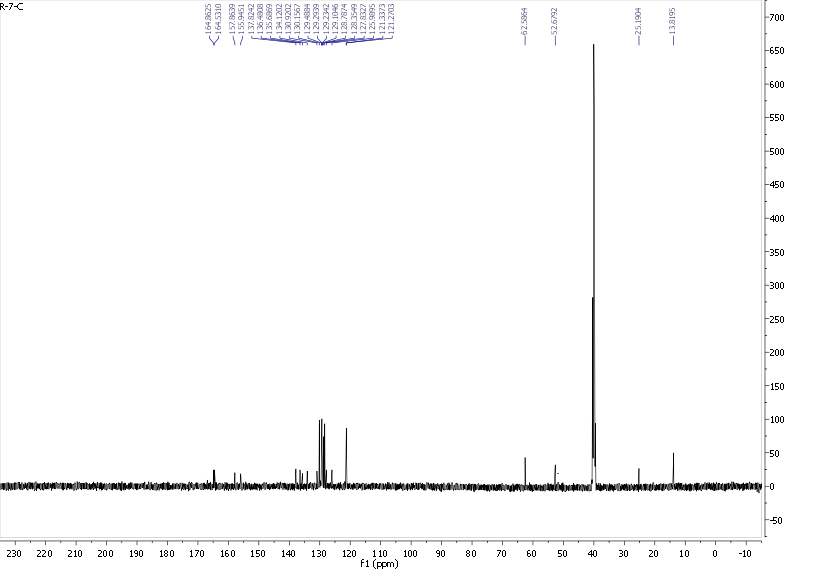

Supplement: Supplementary file 1 — Supplementary Material 1 [file 13065_2023_973_MOESM1_ESM.docx]
